# Comparative phase I randomized open-label pilot clinical trial of Gynophilus^®^ (Lcr regenerans^®^) immediate release capsules versus slow release muco-adhesive tablets

**DOI:** 10.1007/s10096-018-3321-8

**Published:** 2018-07-21

**Authors:** Caroline Dausset, Stéphane Patrier, Pawel Gajer, Claudia Thoral, Yann Lenglet, Jean-Michel Cardot, Philippe Judlin, Jacques Ravel, Adrien Nivoliez

**Affiliations:** 1 Research and Development Department, BIOSE, Aurillac, France; 2 Institute for Genome Sciences, University of Maryland School of Medicine, Baltimore, MD USA; 3 Gynecology and Obstetrics Department, Jacques Lacarin Hospital Center, Vichy, France; 4 Biopharmaceutical Department, UMR MEDIS, Faculty of Pharmacy, University of Clermont Auvergne, Clermont-Ferrand, France; 5 Gynecology and Obstetrics Department, Nancy University Hospital, Nancy, France

**Keywords:** Live biotherapeutic product, Lcr35, Vaginal infections, Vaginal microbiota, Slow release, Muco-adhesive tablet, Safety

## Abstract

Gynophilus^®^ (Lcr regenerans^®^) is a live biotherapeutic product (LBP) that contains the live biotherapeutic microorganism *Lactobacillus rhamnosus* Lcr35^®^, which is indicated to restore vaginal health. The aim of the study was to compare the safety, ease of use, and compliance of two formulations (immediate release: IR capsule and slow release: SR muco-adhesive tablets) as well as the colonization of Lcr35^®^ in healthy women. This phase I study (Comprigel) is a parallel, randomized, 4-arm, and open-label clinical trial evaluating an IR daily capsule formulation vs. a SR tablet administered every 3, 4, or 5 days for 21 days. Self-collected vaginal swabs were used to quantify Lcr35^®^ and characterize the composition and structure of the vaginal microbiota. Both LBPs were well-tolerated, and no severe adverse effects were reported. All groups had Lcr35^®^ vaginal concentrations over 10^7^ colony forming unit per milliliter of vaginal secretion on each day in the study. The new Gynophilus^®^ slow release tablets administered either every 3, 4, or 5 days provided vaginal concentrations that were not significantly different from those of classic Gynophilus^®^ (capsule) once-a-day regimen. The LBPs and the different regimens did not adversely influence the abundance of native *Lactobacillus* spp. and indeed tended to favor their growth and reduce colonization by non-*Lactobacillus* spp. This study illustrates that the SR muco-adhesive LBP tablet (Gynophilus^®^ SR) administered every 3 or 4 days as a safe, well-tolerated, and efficacious alternative to a more demanding IR daily capsule and could protect women’s healthy vaginal microbiome by promoting endogenous *Lactobacillus* spp.

**Electronic supplementary material:**

The online version of this article (10.1007/s10096-018-3321-8) contains supplementary material, which is available to authorized users.

## Introduction

The healthy vaginal microbiota is dominated by *Lactobacillus* spp. which play important roles in protecting women, particularly against vaginal infections [1, 2]. Bacterial vaginosis (BV) and vulvovaginal candidiasis (VVC) are the most prevalent vaginal infections in women of reproductive age [3, 4]. These infections have a negative impact on a woman’s quality of life and represent a significant cost to the healthcare system, especially due to medical consultations and antimicrobial treatments. In addition, recurrence is common and can lead to multiple relapses over time [5, 6]. The use of probiotic products or live biotherapeutic products (LBPs) represent an alternative or complement to traditional treatment regimens to help control and/or re-establish a beneficial vaginal microbiota [7, 8]. Indeed, LBPs are thought to stimulate the recolonization of endogenous *Lactobacillus* spp., which can inhibit the growth of pathogens such as *Gardnerella vaginalis* and *Candida albicans* [7, 8]. LBPs have been used successfully as an adjunct to traditional antibiotics for BV and as an antifungal for VVC to improve the cure rates and prevent recurrence by driving the restoration of beneficial commensal microbiota [3, 9, 10].

LBP Gynophilus immediate release (IR) capsule, containing *Lactobacillus rhamnosus* Lcr35^®^, is commonly recommended for preventive therapies for gynecological indications. Indeed, several clinical trials validated its efficiency for preventing recurrent VVC and BV [11–13].

LBPs were first delivered in the form of vaginal dosage capsules because vaginal administration affects vaginal health more quickly than does oral LBP and enhances the viability of the live biotherapeutic microorganism (LBM) [14]. To be efficacious, a sufficient amount of the probiotic bacteria must be delivered locally. Previous studies have shown that in healthy women, the concentration of *Lactobacillus* spp. is greater than or equal to 10^7^ CFU/ml of vaginal secretion [15, 16]. Capsule administration results in an immediate release of all the LBMs that are linked to the natural vaginal secretions induced by the daily elimination of approximately 10^7^ to 10^8^ CFU per day and therefore requires daily administrations [17]. To limit this rapid loss, Gynophilus^®^ SR tablet was developed to ensure the continuous release and presence of the LBM in sufficient quantities for many days while conserving its in vitro intrinsic characteristics (viability, stability, growth, and pathogen inhibition) [18, 19]. For the first time, a LBM was formulated as slow release tablet for local vaginal administration to enhance women’s quality of life by spacing LBP intake [14, 20].

The objective of this pilot phase I clinical trial was to determine the optimal posology for the new tablet form (SR) compared to the daily capsule (IR). The following criteria were analyzed (i) safety, (ii) ease of use of two galenic forms, (iii) vaginal concentration of the LBM *L. rhamnosus* Lcr35^®^ in healthy women, and (iv) impact of the products on the vaginal microbiota.

## Materials and methods

### Live biotherapeutic products

Two industrial LBPs, provided by BIOSE (Aurillac, France), were used for this study: Gynophilus^®^ (immediate release capsule), and Gynophilus^®^ SR (slow release tablet), both of which were administered vaginally. Gynophilus^®^ capsule contains 350 mg administered daily and the new muco-adhesive Gynophilus^®^ SR vaginal tablet contains 1000 mg. LBPs batch release correspond to *L. rhamnosus* Lcr35^®^ concentration greater than 10^9^ CFU per dosage forms (capsule and tablet). The LBPs and their corresponding characteristics are shown in Table 1. In this article, the LPBs are called by their respective commercial names: Gynophilus^®^ and Gynophilus^®^ SR.

**Table 1:** Industrial LBPs and their characteristics

LBP commercial name	Dosage form	Dosage	LBM	Application
Gynophilus^®^	Capsule	350 mg	*Lcr35* ^®^	Vaginal
Gynophilus^®^ SR	Tablet	1000 mg	*Lcr35* ^®^	Vaginal

### Study design

The study was designed to include at least 32 female volunteers. Following the management of the recruitment centers, the cohort comprised of 35 healthy reproductive age women (> 18 years old). All women were not pregnant (negative urine pregnancy test) and used adapted contraception throughout the trial. Exclusion criteria included, in addition to the classical criteria, gynecological bacterial, fungal, or viral infection in the month prior to or at time of enrollment; use of vaginal or oral probiotics in the last 30 days; or allergy to one of the active ingredients or one of the excipients in the products. Further, additional specific exclusion criteria included an inability to comply with the constraints of the protocol, currently breastfeeding, being post-menopausal (no menstruation in the last 6 months), menstrual bleeding lasting more than 8 days, participated in a clinical study in the last 3 months, severe acute or chronic conditions deemed incompatible with participation in the trial by the clinician, and immunosuppression.

At enrollment, 1 day after the last day of menstruation, each participant completed a medical history questionnaire and was examined by a clinician who collected cervico-vaginal samples to test for *Trichomonas vaginalis, Chlamydia trachomatis, Neisseria gonorrhoeae*, and *Candida* spp. A clinical evaluation was performed at Alfred Fournier Institute according to the Amsel criteria [21]. A second vaginal swab was also collected using the Copan ESwab system (Cat# 480C) by the clinician and stored at BIOSE at − 80 °C in 1 ml of Amies transport medium for *L. rhamnosus* Lcr35^®^ quantification and vaginal microbiota analysis by 16S rRNA gene amplicon sequencing.

Among the 35 volunteer women, one of them had taken a prohibited treatment before the inclusion, and one other woman did not take the test drug after inclusion because of menstruation. Therefore, after obtaining written consent and enrollment, 33 women were randomized to one of four arms: Gynophilus^®^ capsules administered daily which served as the reference arm (REF, *n* = 9) or Gynophilus^®^ SR tablets administered either every 3 (TRT1, *n* = 8), 4 (TRT2, *n* = 9), or 5 (TRT3, *n* = 7) days. Randomization was stratified within enrollment centers (Centre Hospitalier of Aurillac or Vichy, France). The randomization list was prepared using the SAS^®^ software version 9.3.

Women self-administered the first LBPs according the product package insert on the day of enrollment and continued for 21 days according to their assigned regimen. Each day, women self-collected a vaginal swab (Copan ESwab system) and stored the sample at 4 °C in their home fridge. Volunteers delivered all self-collected vaginal swabs to the clinic at the second visit. This second clinical visit occurred within 4 days of the last administration of the assigned LBP. A clinical evaluation similar to that performed at enrollment was performed and a vaginal swab was collected by the clinician using a Copan ESwab. All sample collected were stored at − 80 °C at BIOSE until additional analysis.

### DNA extraction from vaginal samples

An optimized and standardized DNA extraction protocol dedicated to bacterial DNA extraction from swab samples has been used (Genoscreen, Lille, France). ESwabs were thawed on ice and a total of 500 μl of cell suspension in Amies transport medium was processed using the Nucleospin 96 tissue kit (Macherey Nagel, Germany) including a bead-beating step, as recommended by the manufacturer. DNA quantification was performed using SYBR green (Life Technologie, Paisley, UK). A 16S rRNA gene PCR amplification was performed using 2 μl of extracted DNA to confirm the presence of bacterial DNA using standard 16S rRNA gene PCR. DNA extraction and quantification steps were performed by GenoScreen Lille, France.

### Quantification of *L. rhamnosus* Lcr35^®^ by qPCR

Quantification of *L. rhamnosus* Lcr35^®^ was estimated by a specific qPCR run in triplicate as previously described (Darbaky et al. 2016). *L. rhamnosus* Lcr35^®^ quantities were expressed in CFU/ml using a standard curve correlating Ct values to colony forming unit (CFU) from in vitro culture in De Man, Rogosa, and Sharpe (MRS) medium.

### Quantification of total 16S rRNA copies

The number of 16S rRNA gene copies was measured using the BactQuant qPCR assay as previously reported [22]. Estimates of taxa absolute abundance were obtained by multiplying the relative abundance of a specific taxa with the 16S rRNA gene copy number in a sample (Online Resources 1 and 2).

### Vaginal microbiota characterization

The V3-V4 regions of the 16S rRNA gene were amplified from 50 ng of DNA from each sample using the methods of Fadrosh et al. [23] and sequenced on an Illumina MiSeq (Illumina, San Diego, CA, USA) using the 300-bp paired-end protocol. Raw sequence reads were processed using in house scripts and QIIME version 1.6.0 [24] to remove the amplification primer sequences and quality screen was performed according the following criteria: sequence reads with average quality score of less than 20 over a 30-bp sliding window were truncated at the first base pair of the window and evaluated for length. If the trimmed read was < 75% of its original length, it was discarded. Paired sequences were assembled according to Fadrosh et al. [23] and each sequence was assigned to a bacterial taxonomy using the PECAN software [25]. Community state types (CST) according to the previously reported method [26], taxa relative abundance and CST assignments are shown in Online Resources 1 and 2.

### Statistical analysis

Data are presented as the mean and standard deviation for continuous variables and as numbers or proportions (%) for categorical variables. Comparisons between groups were performed by using the Kruskal-Wallis test or Fisher exact test for categorical variables. To compare the mean concentration of Lcr35^®^ over time between treatment and reference arms, the Wilcoxon test was used. All clinical statistical analyses were performed using the SAS software (SAS^®^ institute Cary, NC, USA, www.sas.com); *p* values < 0.05 were considered statistically significant.

Inter-group differences in numbers of 16S rRNA copies and microbiota were assessed using a Kruskal-Wallis test. Theses statistical analyses were carried out using the GraphPad Prism 5 software (www.graphpad.com/prism); *p* values < 0.05 were considered statistically significant.

As a classic pilot study, small arm population induces a low power calculation so the statistical analysis was focused on midpoint and variability.

## Results

### Volunteer population

**Fig. 1:**
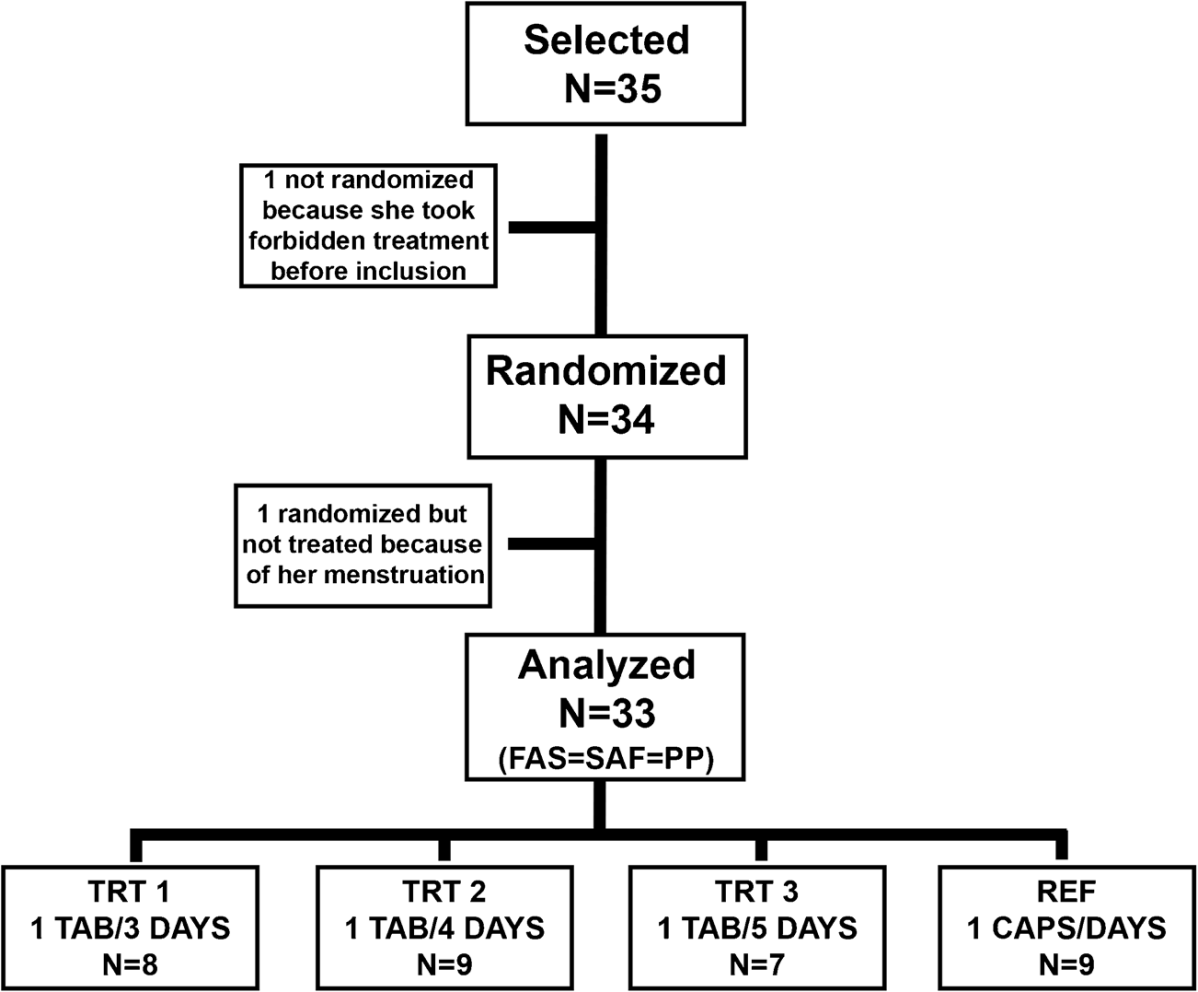
Study design of CompriGel pilot clinical trial

A total of 35 volunteer women were enrolled, and 34 were randomized into 4 treatment arms; 33 women completed the study (Fig. 1). Two women were excluded because of use of treatment before product initiation or menstruation during the treatment.

The population characteristics and statistic results are shown in Table 2. The median participant age was 32 years (32.3 ± 7.7 years). A variability was observed in the median participant’s age among the 4 treatment arms (TRT1, TRT2, TRT3, and REF) (*p* = 0.02). However, all women were between 21 and 52 years of age. Body mass index (BMI) was not significantly different (*p* = 0.60) between each arm, with a mean of 23 ± 5 (kg/m^2^). Overall, 54.5% of the participants had at least one previous live birth. The mean number of children was 2 for the entire cohort, varying from 1 in TRT2 to 3 in TRT1 (Table 2).

**Table 2:** Demographic characteristics of the population—FAS population (*N* = 33^Δ^)

Parameter	Statistics	TRT1 (1 tab/3 days) (N = 8)	TRT2 (1 tab/4 days) (N = 9)	TRT3 (1 tab/5 days) (N = 7)	REF (1 caps/ day) (N = 9)	*p* (< 0.05)
Age at inclusion (years)	*Mean ± SD*	39.4 ± 6.5	28.9 ± 4.8	32.4 ± 9.7	29.4 ± 5.7	0.0220**
Body mass index (kg/m^2^)	*Mean ± SD*	24.53 ± 4.95	22.67 ± 6.19	22.47 ± 3.21	22.44 ± 4.29	0.5988**
Children						
No	*n (%)*	2 (25.0%)	5 (55.6%)	3 (42.9%)	5 (55.6%)	0.6115*
Yes	*n (%)*	6 (75.0%)	4 (44.4%)	4 (57.1%)	4 (44.4%)	
If yes, number of children	*Mean ± SD*	2.7 ± 0.5	1.0 ± 0.0	2.0 ± 0.8	1.5 ± 0.6	0.0652**

*Fisher’s exact test

**Kruskal-Wallis test

^Δ^*N* = 33 because only 33 received the treatment

### Safety of Lcr regenerans^®^ products

No severe adverse events were observed (Table 3). Ten adverse events (AE) were reported, but no differences (*p* = 0.40) in the frequency of AEs were detected among the 4 arms (Table 3). All AEs were considered not serious. One of the AEs in TRT2 was deemed to be of severe intensity with vulvar itching. Other reported AEs included three that were linked to product use, and the women reported vulvar itching, brown discharges, and vulvovaginal discomfort after administration of the first tablet. However, none of the AEs led to permanent or temporary withdrawal from the study (Table 3).

**Table 3:** Safety analysis—adverse events during the treatment period (*N* = 33^Δ^)

Volunteer with at least one	TRT1 (1 tab/3 days) (N = 8)	TRT2 (1 tab/4 days) (N = 9)	TRT3 (1 tab/5 days) (N = 7)	REF (1 caps/ day) (N = 9)	Total (N = 33)	p*(< 0.05)
SAE (*n*)	0	0	0	0	0	NA
AE (*n*)	4	1	2	3	10	0.4017
AE of severe intensity (*n*)	0	1	0	0	0	NA
AE linked to administration of the trial treatment (*n*)	1	1	1	0	3	0.7773
AE that led to permanent or temporary discontinuation of study treatment (*n*)	0	0	0	0	0	NA

*Fisher’s exact test

^Δ^*N* = 33 because only 33 received the treatment

### Tolerance/acceptance

Clinical tolerance was evaluated at the last visit by the study clinician through questioning and examination of the participants. Factors evaluated were onset of clinical signs and/or urogenital symptoms (Table 4). The products were well-tolerated (score: excellent) by half the subjects and were tolerated in the other half (score: good). Specific criteria evaluation (tingling, dryness, burning, itching, and pelvic pain) was rarely reported, and no difference in these reported factors between arms was observed (Table 5).

**Table 4:** Overall tolerance (*N* = 33^Δ^)

Volunteer evaluation	TRT1 (1 tab/3 days) (N = 8)	TRT2 (1 tab/4 days) (N = 9)	TRT3 (1 tab/5 days) (N = 7)	REF (1 caps/day) (N = 9)	Total (N = 33)	p* (< 0.05)
Very good tolerance	4	6	2	4	16	0.5446
Good tolerance	4	3	5	4	16	
Average tolerance	0	0	0	0	0	
Poor tolerance	0	0	0	0	0	

*Fisher’s exact test

^Δ^*N* = 33 because only 33 received the treatment

**Table 5:** Satisfaction of female volunteers with regard to treatment tolerance

Parameter	Statistics	TRT1 (1 tab/3 days) (N = 8)	TRT2 (1 tab/4 days) (N = 9)	TRT3 (1 tab/5 days) (N = 7)	REF (1 caps/day) (N = 9)	Total (N = 33)	Global p value** (< 0.05)
Tingling	*Mean ± SD*	0.43 ± 0.69	1.13 ± 3.18	0.67 ± 1.43	0.28 ± 0.22	0.63 ± 1.77	*0.5856*
0 = no tingling							
10 = severe tingling							
Dryness	*Mean ± SD*	0.30 ± 0.38	0.11 ± 0.15	0.50 ± 0.98	0.29 ± 0.19	0.29 ± 0.50	*0.3568*
0 = no dryness							
10 = severe dryness							
Burning	*Mean ± SD*	0.29 ± 0.38	0.14 ± 0.25	0.14 ± 0.18	0.23 ± 0.19	0.20 ± 0.26	*0.5970*
0 = no burning							
10 = severe burning							
Itching	*Mean ± SD*	0.66 ± 0.86	1.22 ± 3.26	0.97 ± 2.35	1.37 ± 3.24	1.07 ± 2.56	*0.5747*
0 = no itching							
10 = severe itching							
Pelvic pain	*Mean ± SD*	0.29 ± 0.34	1.18 ± 2.41	0.11 ± 0.15	0.48 ± 0.82	0.55 ± 1.35	*0.6258*
0 = no pelvic pain							
10 = severe pelvic pain							

**Kruskal-Wallis test

Administration and dosage regimen demonstrated strong satisfaction (Table 6). The tablet regimen showed significantly higher acceptance after the 3-week administration, especially in the less restrictive arms, in which the tablets were taken every 5 days compared to the daily capsule (Table 6).

**Table 6:** Satisfaction of female volunteers with regard to administration and dosage regimen

Parameter	Statistics	TRT1 (1 tab/3 days) (N = 8)	TRT2 (1 tab/4 days) (N = 9)	TRT3 (1 tab/5 days) (N = 7)	REF (1 caps/day) (N = 9)	Total (N = 33)	Global p value** (< 0.05)
Administration of the treatment	*Mean ± SD*	0.84 ± 0.61	1.04 ± 1.11	1.00 ± 1.20	1.00 ± 1.20	0.92 ± 0.86	*0.9740*
0 = easy administration							
10 = difficult administration							
*Size of the* administered capsules/tablets	*Mean ± SD*	1.33 ± 2.45	1.08 ± 0.97	1.61 ± 2.50	0.58 ± 0.36	1.12 ± 1.70	*0.5983*
0 = an appropriate size							
10 = an inappropriate size							
Use of intravaginal administration of gynecological-related treatments	*Mean ± SD*	0.91 ± 0.93	2.01 ± 1.59	1.43 ± 1.41	1.99 ± 1.82	1.62 ± 1.50	*0.2635*
0 = is practical							
10 = is restrictive							
Administration of capsules/tablets	*Mean ± SD*	0.59 ± 0.72	1.52 ± 1.66	0.79 ± 0.54	1.12 ± 1.12	1.03 ± 1.14	*0.5565*
0 = an appropriate shape							
10 = not practical							
Use of the capsule/tablet	*Mean ± SD*	0.84 ± 0.79	2.50 ± 1.66	1.47 ± 1.18	3.09 ± 2.82	2.04 ± 1.97	*0.1381*
0 = is ideal							
10 = is restrictive							
Administration of a capsule every day/every 3, 4, and 5 days	*Mean ± SD*	2.33 ± 3.48	2.61 ± 2.38	0.96 ± 1.46	5.38 ± 3.35	2.95 ± 3.15	*0.0270*
0 = is not restrictive							
10 = is restrictive							
The 3-week period of administration	*Mean ± SD*	2.94 ± 3.32	2.39 ± 1.41	1.27 ± 1.53	5.61 ± 2.95	3.16 ± 2.86	*0.0364*
0 = is not restrictive							
10 = is restrictive							
Release of the capsule/tablet	*Mean ± SD*	3.20 ± 2.83	4.16 ± 3.63	3.49 ± 3.06	2.88 ± 2.72	3.43 ± 2.98	*0.8958*
0 = complete “disintegration” of the capsule/tablet							
10 = no “disintegration” of the administered capsule/tablet which was found completely expelled from the vagina							

**Kruskal-Wallis test

### Lcr35^®^ colonization

**Fig. 2:**
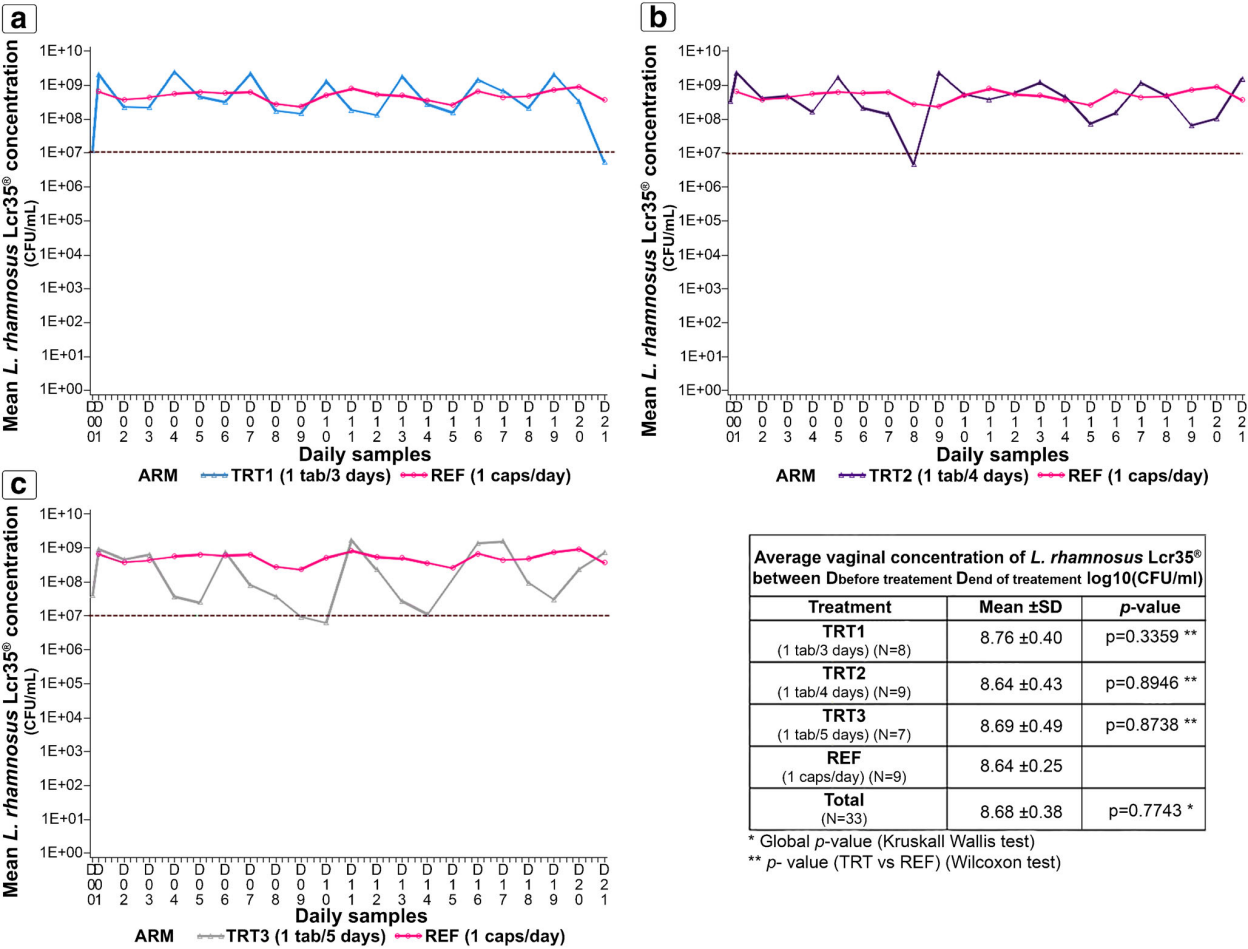
Mean vaginal concentrations of *Lactobacillus rhamnosus* Lcr35^®^ over time in each arm measured by the targeted qPCR method. Delivery of 1 tablet every 3 days (**a**), 1 tablet every 4 days (**b**), and 1 tablet every 5 days (**c**) were compared to the reference treatment of 1 capsule per day

Women self-collected a daily vaginal swab that was used to measure the concentration of *Lactobacillus rhamnosus* Lcr35^®^ in each arm (Fig. 2). The average vaginal Lcr35^®^ concentration showed no major difference among the four arms (*p* = 0.77). A decrease in the daily Lcr35^®^ concentration was observed post-tablet administration as the frequency of administration increased from every 3 to every 5 days (Fig. 2). Although the daily Lcr35^®^ concentration decreased post-administration, it remained greater than 10^7^ CFU/ml in TRT1 and TRT2, with the exception of 1 day in TRT2. In TRT3, 5 days post-administration, the Lcr35^®^ concentration was approximately 10^7^ CFU/ml.

### Effect of Gynophilus^®^ formulations on the vaginal microbiota

**Fig. 3:**
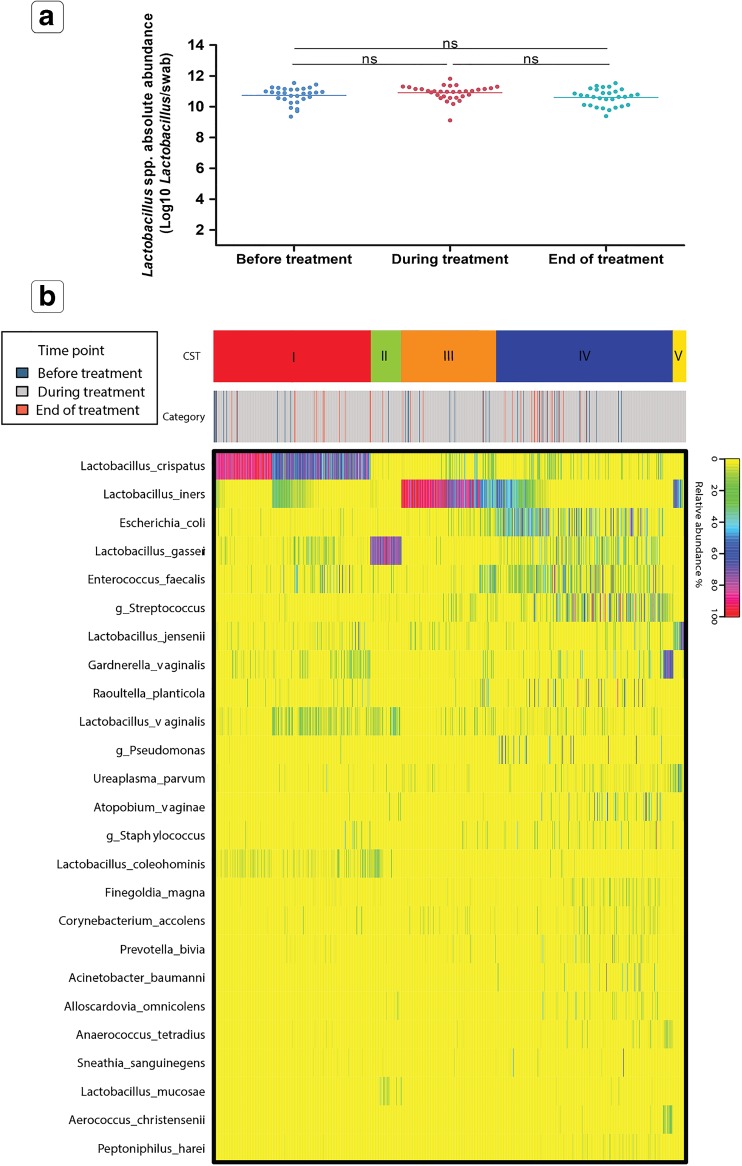
Composition of the vaginal microbiota in all treatment arms. **a** Effect of all treatments combined on the estimates of *Lactobacillus* spp. absolute abundance at visit 1, during treatment and at visit 2. **b** Community state type distribution over all samples collected in the study based on the species composition and abundance, as determined by the heatmap of percentage abundance of microbial taxa found in the vaginal microbial communities of all samples

The daily composition and absolute abundance of the vaginal microbiota were evaluated using 16S rRNA gene amplicon sequencing and quantitative PCR. No significant difference (*p* = 0.12) in the concentration of non-Lcr35^®^ *Lactobacillus* spp. was observed before treatment (D0), during treatment and at the end of treatment (D21) (Fig. 3a). The overall composition in *Lactobacillus* spp. remained consistent during and after treatment.

The vaginal microbiota was classified into six distinct community state types (CSTs) based on the microbiota bacterial composition and abundance. The before and after treatment CST frequencies are shown in Table 7, where CST-I is dominated by *L. crispatus*, CST-II is dominated by *L. gasseri*, CST-III is dominated by *L. iners*, CST-V is dominated by *L. jensenii*, CST-IV is characterized by a paucity of *Lactobacillus* spp. and a diverse set of strict and facultative anaerobes, and CST-VI is dominated by the LBM Lcr35^®^. The frequency of CSTs was not different between visit 1 and visit 2, indicating that Gynophilus^®^ treatments did not disturb the indigenous vaginal microbiota. Only one on 35 women is classified as CST-VI (dominance Lcr35^®^) because the last tablet was administrated just before the V2. So, Lcr35^®^ did not colonize and replace the endogenous microbiota. Moreover, the CST proportions were not drastically modified which suggest that Lcr35^®^ did not promote only one CST type.

**Table 7:** CST frequency at visit 1 and visit 2 (*N* = 33)

		CST-I	CST-II	CST-III	CST-IV	CST-V	CST-VI
Visit 1 (D0)	*n (%)*	10 (30.3%)	2 (6.1%)	6 (18.2%)	15 (45.4%)	0 (0.0%)	0 (0.0%)
Visit 2 (D21)	*n (%)*	10 (30.3%)	2 (6.1%)	4 (12.1%)	16 (48.5%)	0 (0.0%)	1 (3.0%)

**Fig. 4:**
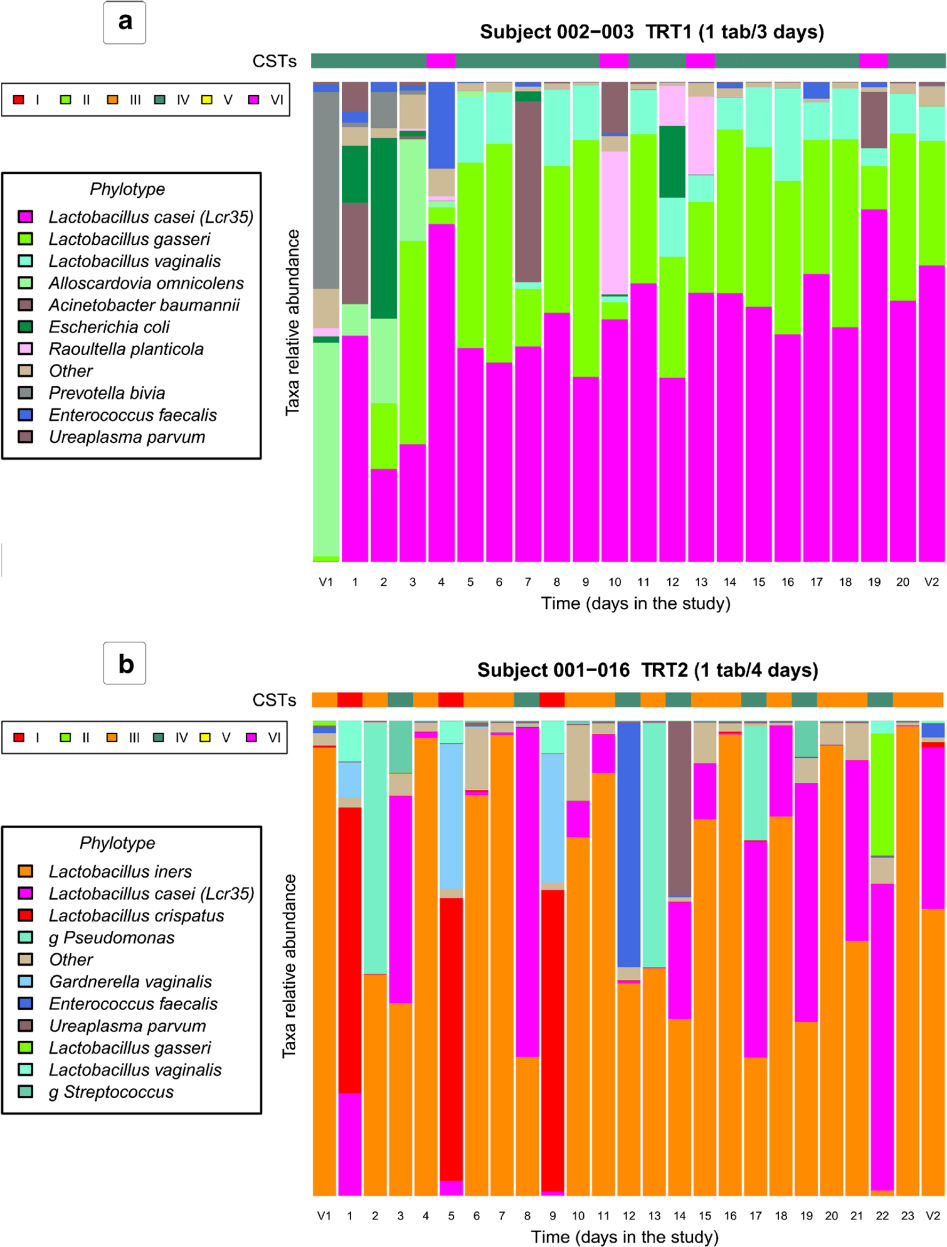
Representative longitudinal vaginal microbiota profiles of women enrolled in **a** treatment 1; **b** treatment 2. *Lactobacillus casei* (pink) indicates the live biotherapeutic microorganism Lcr35^®^. Vertically, each column represents one sampling day. Horizontally, community state type (CST) distribution for all samples collected by women was determined by the abundance of phylotype composition represented in the taxa relative abundance histogram. CST reflect the dominant microorganisms, CST-I is dominated by *L. crispatus*, CST-II by *L. gasseri*, CST-III by *L. iners*, CST-V by *L. jensenii,* CST-VI by *L. casei (Lcr35*^®^), and CST-IV is characterized by a diverse set of strict and facultative anaerobes. The vaginal microbiota study of each volunteer women is presented in Online Resource 3

Representative vaginal microbiota profiles for each study arm and the absolute abundance of Lcr35^®^ are shown in Fig. 4 and highlight the absence of a rapid change in the composition and structure of the vaginal microbiota during treatment. Of note, it was observed that the *Escherichia coli* present at visit 1 was rapidly eliminated after the initiation of treatment and that the indigenous *Lactobacillus* spp. re-colonized the vagina (Fig. 4a). Moreover, *E. coli* species was detected at least once during the study in vaginal samples of 28 women. For 21 of them, *E. coli* is eliminated quickly after treatment (Online Resource 3). Overall, after the initiation of Gynophilus^®^ treatments, *Lactobacillus* spp. abundance remained stable or promoted (Fig. 4).

## Discussion

LBPs aim to re-establish a *Lactobacillus*-dominated vaginal microbiota by promoting the recolonization of the indigenous *Lactobacillus* spp., thereby restoring the protection against infections [7, 8, 27, 28]. The LBP Gynophilus^®^ is vaginally delivered and is indicated to prevent recurrent VVC and BV by restoring vaginal health [11–13]. This LBP contains *L. rhamnosus* Lcr35^®^, which was previously reported to have potent antimicrobial properties [18, 19, 29–32]. Gynophilus^®^ (Lcr regenerans^®^) LBP formulated in vaginal capsules was shown to be well-tolerated and significantly enhanced the quality of life of women after treatment [13]. A muco-adhesive slow release tablet formulation (SR) was developed to limit the rapid loss of the LBP associated with natural vaginal secretions and ultimately enhance the ease of use by minimizing the frequency of administration [14, 18–20] resulting in an increased compliance. CompriGel is a pilot phase I clinical trial, in which 35 volunteers were randomized to four arms to compare the LBP vaginal concentrations between daily IR capsule administration and SR tablets taken every 3, 4, or 5 days for 21 days. As most of the pilot clinical studies, the power calculation is limited due to the small size population but minimize the risks in healthy subjects while allowing selection of an interesting posology. So, this pilot study was designed to determine the more adapted posology of the new SR tablet.

In this study, we have shown no difference in Lcr35^®^ colonization between SR tablets administered every 3 or 4 days and daily capsules. Importantly, the mean concentration of *L. rhamnosus* Lcr35^®^ in all treatment arms remained greater than 10^7^ CFU/ml, which is the necessary minimal concentration for activity and, ultimately, to prevent vaginal infections [17]. The Lcr35^®^ colonization was quantified by molecular biology. This method was not used to differentiate viable and dead microorganisms and we know that the delivered LBM concentration is at least 1.10^9^ CFU/posology and we observe a quickly remove of the LBM probably induced by the vaginal secretions [17]. So, administration of tablets every 3 or 4 days maintained a mean Lcr35^®^ concentration greater than 10^9^ CFU/ml, similar to that obtained with the daily capsules. This finding supports the possibility of a clinical regimen in which Gynophilus^®^ SR tablets are administered every 3 or 4 days while maintaining maximum efficacy.

The formulation of the muco-adhesive Gynophilus^®^ SR tablet was developed to provide a three-fold increase in the quantity of LBM brought in one dosage form to reduce the rapid loss of LBM Lcr35^®^ delivered by a capsule. Moreover, this new dosage form ultimately improved the comfort and ease of use, as the controlled muco-adhesive vaginal formulation affords a prolonged residence time of the LBM at efficacious concentrations with less frequent administration. Slow release delivery strategies have been successfully developed for antifungal and antibacterial drugs and for other vaginal LBPs using different muco-adhesive tablets or vaginal rings [33–37].

The main goal of slow release strategies is to promote compliance when the recommended treatment duration is longer than 2–3 days and, ultimately, to improve the comfort and ease of use by reducing the burden on women through spacing administrations. The results presented have confirmed that over a 3-week treatment period, Gynophilus^®^ SR tablets administered every 3 or 4 days were reported to be convenient and less burdensome than were daily Gynophilus^®^ capsules (Table 6). Daily treatment schedules generally induce poor compliance over long administration periods. Gynophilus^®^ SR promoted compliance and improved treatment adherence. This finding supports a benefit of the LBP for treating recurrent vulvovaginal infections, which have been shown to require a 3-week treatment [13]. While compliance and ease of use are important factors, the safety of the product is of the utmost importance. In this phase I clinical trial, no severe adverse events (AE) and only a few AEs were reported; these AEs were reported equally in all arms of the study. None of the AEs led to temporary discontinuation or withdrawal from the study. Overall, the tolerance of the LBPs was reported to be excellent in half of the participants and good in the other half and was associated with an overall strong satisfaction with the use of the products. These results are in line with previous clinical trials for the prevention of recurrent VVC and BV with various Gynophilus^®^ formulations [11, 13]. Gynophilus^®^ SR appears to be a well-accepted, safe, and easy-to-use LBP with high compliance that delivers an adequate concentration of LBM Lcr35^®^ for up to 5 days post-administration. The efficacy of Gynophilus^®^ SR must be confirmed in a large-scale study with the final selected dose.

The impact of Gynophilus^®^ LBP formulation and treatment frequency on the composition and structure of the vaginal microbiota was evaluated. The results showed that at study entry, 54.6% of the volunteer’s vaginal microbiota harbored microorganisms that were phylogenetically related to *Lactobacillus* spp. as the numerically dominant members (Table 7). The frequency of vaginal CSTs in these otherwise healthy French women is similar to that observed by Ravel et al. in North American women [26, 38]. In this phase I clinical study, Gynophilus^®^ capsules and SR tablet administration did not disturb the endogenous *Lactobacillus* spp. and trends towards an increase in the abundance of indigenous *Lactobacillus* spp. without Lcr35^®^ colonization (Fig. 3 and Fig. 4). This finding supports a beneficial effect of treatment by restoring a healthy *Lactobacillus-*dominated vaginal microbiota. However, because all participants are healthy and disease free, a clinical trial designed to evaluate treatment efficacy in women with VVC or BV is warranted to confirm that Gynophilus^®^ SR administered every 3 or 4 days will also promote the restoration of a resilient *Lactobacillus* spp.-dominated microbiota while efficiently treating the vaginal infections.

Even if statistical results showed no differences between both galenic forms, it has to be remind that low number of subject does not allow any extrapolation to the global population. This pilot clinical trial will afford statistical power calculation to design a larger and more robust clinical trial to confirm the Gynophilus^®^ SR efficacy on vaginal infections.

In conclusion, this study showed that the SR formulation of Gynophilus^®^ administered every 3, 4, or 5 days is well-tolerated, easy-to-use, and less burdensome than the capsule form. More importantly, the findings support the conclusion that the muco-adhesive Gynophilus^®^ SR administered every 3, 4, or 5 days is a safe, well-tolerated, well-accepted, and efficient alternative to provide a daily vaginal concentration of LBM greater than 10^7^ CFU/mL while maintaining and even favoring a woman’s indigenous *Lactobacillus* spp. Thus, Gynophilus^®^ SR administered every 3 or 4 days is an efficacious alternative for patients with poor compliance profiles.

## Electronic supplementary material

ESM 1 (XLSX 368 kb)

ESM 2 (XLSX 235 kb)

ESM 3 (PDF 2197 kb)

## References

[1] Charbonneau MR, Blanton LV, DiGiulio DB (2016). Human developmental biology viewed from a microbial perspective. Nature.

[2] Belizário JE, Napolitano M (2015). Human microbiomes and their roles in dysbiosis, common diseases, and novel therapeutic approaches. Front Microbiol.

[3] Bautista CT, Wurapa E, Sateren WB (2016). Bacterial vaginosis: a synthesis of the literature on etiology, prevalence, risk factors, and relationship with chlamydia and gonorrhea infections. Mil Med Res.

[4] Sobel JD, Faro S, Force RW (1998). Vulvovaginal candidiasis: epidemiologic, diagnostic, and therapeutic considerations. Am J Obstet Gynecol.

[5] Hanson L, VandeVusse L, Jermé M (2016). Probiotics for treatment and prevention of urogenital infections in women: a systematic review. J Midwifery Womens Health.

[6] Rathod SD, Buffler PA (2014). Highly-cited estimates of the cumulative incidence and recurrence of vulvovaginal candidiasis are inadequately documented. BMC Womens Health.

[7] Parma M, Stella Vanni V, Bertini M, Candiani M (2014). Probiotics in the prevention of recurrences of bacterial vaginosis. Altern Ther Health Med.

[8] Vuotto C, Longo F, Donelli G (2014). Probiotics to counteract biofilm-associated infections: promising and conflicting data. Int J Oral Sci.

[9] Bilardi J, Walker S, McNair R (2016). Women’s management of recurrent bacterial vaginosis and experiences of clinical care: a qualitative study. PLoS One.

[10] Marshall AO (2015). Managing recurrent bacterial vaginosis: insights for busy providers. Sex Med Rev.

[11] Kovachev SM, Vatcheva-Dobrevska RS (2015). Local probiotic therapy for vaginal Candida albicans infections. Probiotics Antimicrob Proteins.

[12] Petricevic L, Witt A (2008). The role of Lactobacillus casei rhamnosus Lcr35 in restoring the normal vaginal flora after antibiotic treatment of bacterial vaginosis. BJOG Int J Obstet Gynaecol.

[13] Kern AM, Bohbot JM, Cardot JM (2012) Traitement préventif de la candidose vulvovaginale récidivante par probiotique vaginal: résultats de l’étude observationnelle Candiflore. Lett Gynécologue 34–37

[14] Vicariotto F, Del Piano M, Mogna L, Mogna G (2012). Effectiveness of the association of 2 probiotic strains formulated in a slow release vaginal product, in women affected by vulvovaginal candidiasis: a pilot study. J Clin Gastroenterol.

[15] Boskey ER, Telsch KM, Whaley KJ (1999). Acid production by vaginal flora in vitro is consistent with the rate and extent of vaginal acidification. Infect Immun.

[16] Bartlett JG, Onderdonk AB, Drude E (1977). Quantitative bacteriology of the vaginal flora. J Infect Dis.

[17] Mitchell C, Paul K, Agnew K (2011). Estimating volume of cervicovaginal secretions in cervicovaginal lavage fluid collected for measurement of genital HIV-1 RNA levels in women. J Clin Microbiol.

[18] Nivoliez A, Camares O, Paquet-Gachinat M (2012). Influence of manufacturing processes on in vitro properties of the probiotic strain Lactobacillus rhamnosus Lcr35®. J Biotechnol.

[19] Muller C, Mazel V, Dausset C (2014). Study of the Lactobacillus rhamnosus Lcr35® properties after compression and proposition of a model to predict tablet stability. Eur J Pharm Biopharm.

[20] Palmeira-de-Oliveira R, Palmeira-de-Oliveira A, Martinez-de-Oliveira J (2015). New strategies for local treatment of vaginal infections. Adv Drug Deliv Rev.

[21] Amsel R, Totten PA, Spiegel CA (1983). Nonspecific vaginitis. Diagnostic criteria and microbial and epidemiologic associations. Am J Med.

[22] Liu CM, Aziz M, Kachur S (2012). BactQuant: an enhanced broad-coverage bacterial quantitative real-time PCR assay. BMC Microbiol.

[23] Fadrosh DW, Ma B, Gajer P (2014). An improved dual-indexing approach for multiplexed 16S rRNA gene sequencing on the Illumina MiSeq platform. Microbiome.

[24] Caporaso JG, Kuczynski J, Stombaugh J (2010). QIIME allows analysis of high-throughput community sequencing data. Nat Methods.

[25] Holm JB, Gajer P, Ravel J (2016) PECAN A fast, novel 16S rRNA gene sequence non-clustering based taxonomic assignment tool. 16th Int Symp Microb Ecol Montr Can

[26] Ravel J, Gajer P, Abdo Z (2011). Vaginal microbiome of reproductive-age women. Proc Natl Acad Sci.

[27] Anukam KC, Osazuwa E, Osemene GI (2006). Clinical study comparing probiotic lactobacillus GR-1 and RC-14 with metronidazole vaginal gel to treat symptomatic bacterial vaginosis. Microbes Infect.

[28] Falagas ME, Betsi GI, Athanasiou S (2006). Probiotics for prevention of recurrent vulvovaginal candidiasis: a review. J Antimicrob Chemother.

[29] Nivoliez A, Veisseire P, Alaterre E (2015). Influence of manufacturing processes on cell surface properties of probiotic strain Lactobacillus rhamnosus Lcr35. Appl Microbiol Biotechnol.

[30] Dapoigny M, Piche T, Ducrotte P (2012). Efficacy and safety profile of LCR35 complete freeze-dried culture in irritable bowel syndrome: a randomized, double-blind study. World J Gastroenterol: WJG.

[31] Bohbot JM, Cardot JM (2012). Vaginal impact of the oral administration of total freeze-dried culture of LCR 35 in healthy women. Infect Dis Obstet Gynecol.

[32] Coudeyras S, Marchandin H, Fajon C, Forestier C (2008). Taxonomic and strain-specific identification of the probiotic strain Lactobacillus rhamnosus 35 within the Lactobacillus casei group. Appl Environ Microbiol.

[33] Gunawardana M, Mullen M, Yoo J (2014). Sustained delivery of commensal bacteria from pod-intravaginal rings. Antimicrob Agents Chemother.

[34] Baloglu E, Ay Senyıgıt Z, Karavana SY (2011). In vitro evaluation of mucoadhesive vaginal tablets of antifungal drugs prepared with thiolated polymer and development of a new dissolution technique for vaginal formulations. Chem Pharm Bull (Tokyo).

[35] Perioli L, Ambrogi V, Pagano C (2009). FG90 chitosan as a new polymer for metronidazole mucoadhesive tablets for vaginal administration. Int J Pharm.

[36] Nader-Macias MEF, de Ruiz CS, Ocana VS, Juarez Tomas MS (2008). Advances in the knowledge and clinical applications of lactic acid bacteria as probiotics in the urogenital tract. Curr Womens Health Rev.

[37] Maggi L, Mastromarino P, Macchia S (2000). Technological and biological evaluation of tablets containing different strains of lactobacilli for vaginal administration. Eur J Pharm Biopharm.

[38] Hickey RJ, Zhou X, Pierson JD (2012). Understanding vaginal microbiome complexity from an ecological perspective. Transl Res.

