# Supplementary material for: Comparative phase I randomized open-label pilot clinical trial of Gynophilus® (Lcr regenerans®) immediate release capsules versus slow release muco-adhesive tablets
Source: Eur J Clin Microbiol Infect Dis. 2018 Jul 21;37(10):1869–80. doi: 10.1007/s10096-018-3321-8 (PMC6154122; doi:10.1007/s10096-018-3321-8)
Supplement: Supplementary file 3 — (PDF 2197 kb) [file 10096_2018_3321_MOESM3_ESM.pdf]

Comparative phase I randomized open-label pilot clinical trial of Gynophilus® (Lcr regenerans®) Immediate Release capsules versus Slow Release muco-adhesive tablets.

European Journal of Clinical Microbiology & Infectious Diseases

Caroline DAUSSET[a#], Stéphane PATRIER[a], Pawel GAJER[b], Claudia THORAL[a], Yann LENGLET[c], Jean-Michel CARDOT[d], Philippe JUDLIN[e], Jacques RAVEL[b], Adrien NIVOLIEZ[a]

[a] Research and development department, BIOSE, Aurillac, France

[b] Institute for Genome Sciences, University of Maryland School of Medicine, Baltimore, Maryland, USA

[c] Gynecology and obstetrics department, Jacques Lacarin Hospital Center, Vichy, France

[d] Biopharmaceutical Department, UMR MEDIS, Faculty of Pharmacy, University of Clermont Auvergne, Clermont-Ferrand, France

[e] Gynecology and Obstetrics Department, Nancy University Hospital, Nancy, France

# Corresponding author, c.dausset@biose.com

Online Resource 3: Representative longitudinal vaginal microbiota profiles of women enrolled in each of the treatment arms ((a) treatment 1; (b) treatment 2; (c) treatment 3; (d) reference treatment). *Lactobacillus casei* (pink) indicates the live biotherapeutic microorganism Lcr35®. Vertically, each column represents one sampling day. Horizontally, community state type (CST) distribution for all samples collected by women was determined by the abundance of phylotype composition represented in the taxa relative abundance histogram. CST reflect the dominant microorganisms, CST-I is dominated by *L. crispatus*, CST-II by *L. gasseri*, CST-III by *L. iners*, CST-V by *L. jensenii*, CST-VI by *L. casei (Lcr35®)* and CST-IV is characterized by a diverse set of strict and facultative anaerobes.

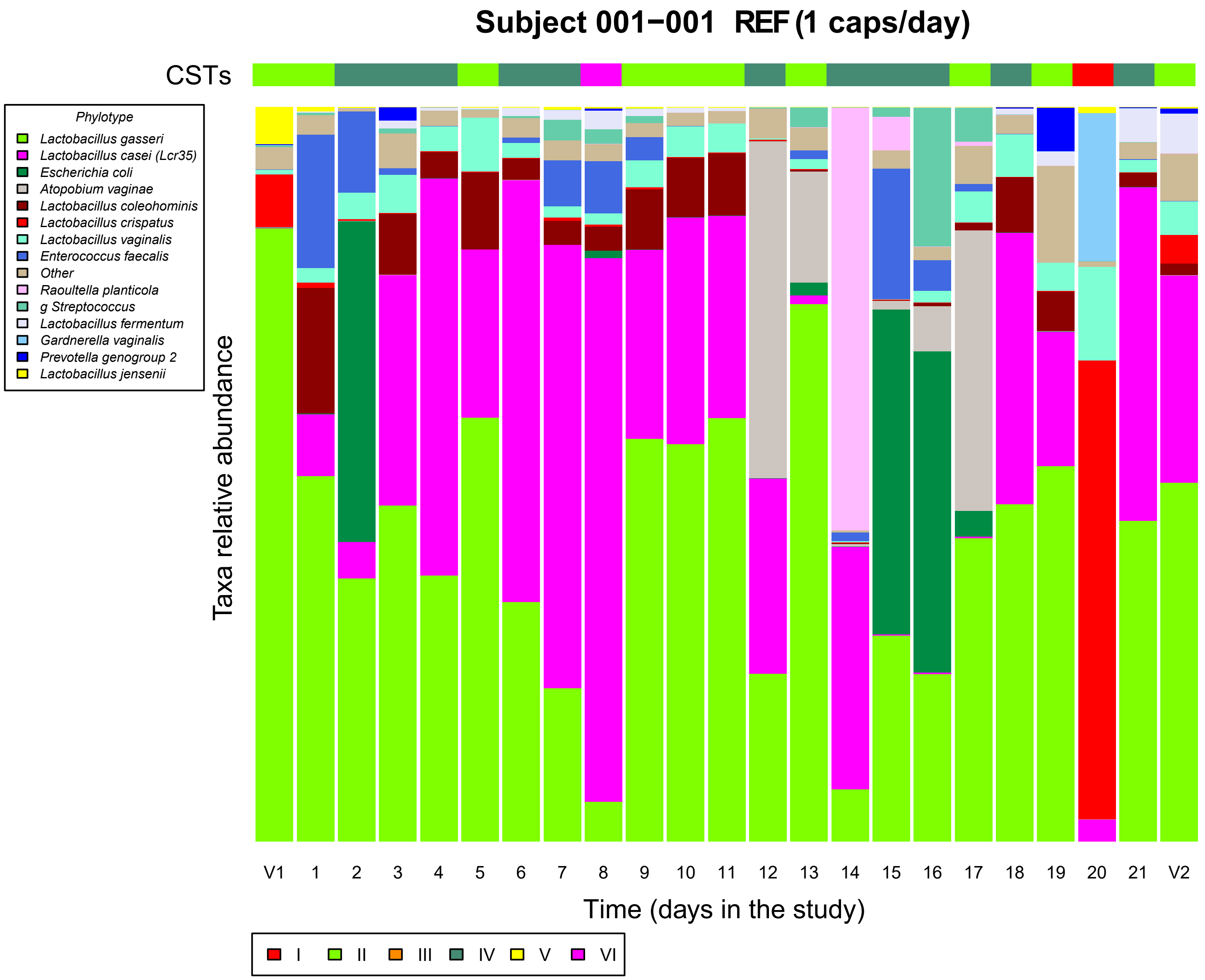

Subject 001−001 REF (1 caps/day)
CSTs
Phylotype
Lactobacillus gasseri
Lactobacillus casei (Lcr35)
Escherichia coli
Atopobium vaginae
Lactobacillus coleohominis
Lactobacillus crispatus
Lactobacillus vaginalis
Enterococcus faecalis
Other
Raoultella planticola
g Streptococcus
Lactobacillus fermentum
Gardnerella vaginalis
Prevotella genogroup 2
Lactobacillus jensenii
Taxa relative abundance
V1 1 2 3 4 5 6 7 8 9 10 11 12 13 14 15 16 17 18 19 20 21 V2
Time (days in the study)
I II III IV V VI

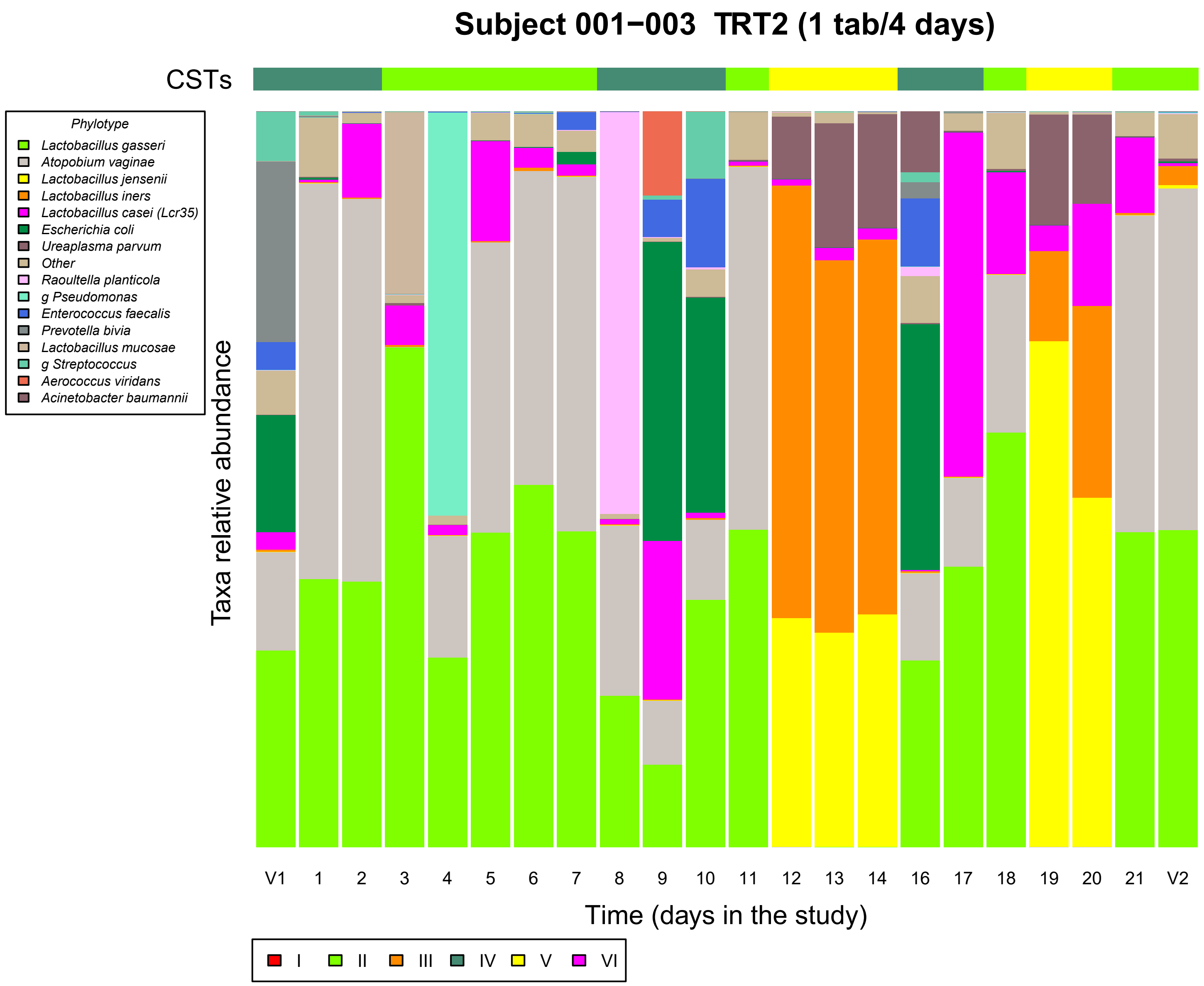
Subject 001−003 TRT2 (1 tab/4 days)
CSTs
Phylotype
Lactobacillus gasseri
Atopobium vaginae
Lactobacillus jensenii
Lactobacillus iners
Lactobacillus casei (Lcr35)
Escherichia coli
Ureaplasma parvum
Other
Raoultella planticola
g Pseudomonas
Enterococcus faecalis
Prevotella bivia
Lactobacillus mucosae
g Streptococcus
Aerococcus viridans
Acinetobacter baumannii
Taxa relative abundance
V1 1 2 3 4 5 6 7 8 9 10 11 12 13 14 16 17 18 19 20 21 V2
Time (days in the study)
I II III IV V VI

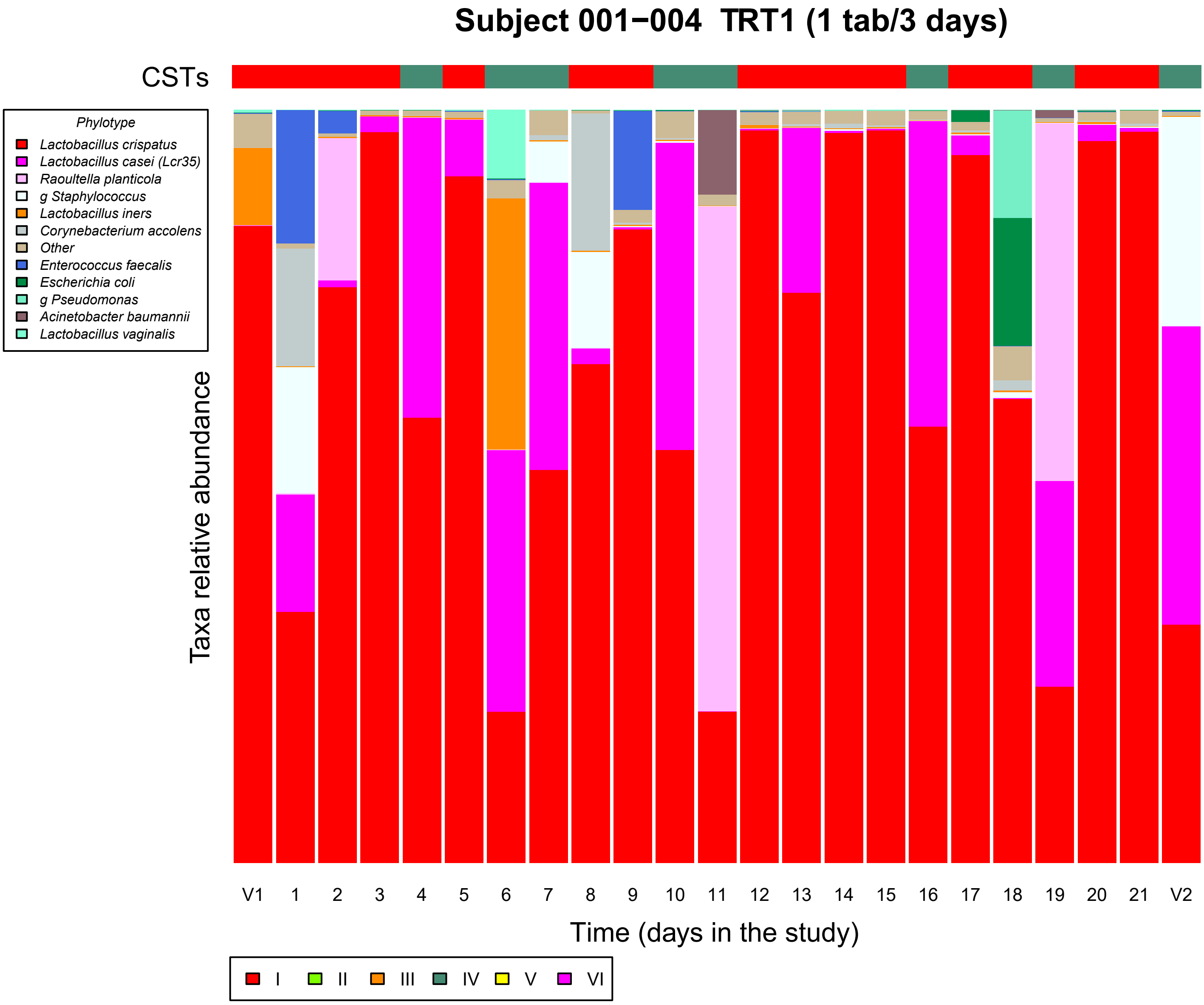

Subject 001−004 TRT1 (1 tab/3 days)
CSTs
Phylotype
Lactobacillus crispatus
Lactobacillus casei (Lcr35)
Raoultella planticola
g Staphylococcus
Lactobacillus iners
Corynebacterium accolens
Other
Enterococcus faecalis
Escherichia coli
g Pseudomonas
Acinetobacter baumannii
Lactobacillus vaginalis
Taxa relative abundance
V1 1 2 3 4 5 6 7 8 9 10 11 12 13 14 15 16 17 18 19 20 21 V2
Time (days in the study)
I II III IV V VI

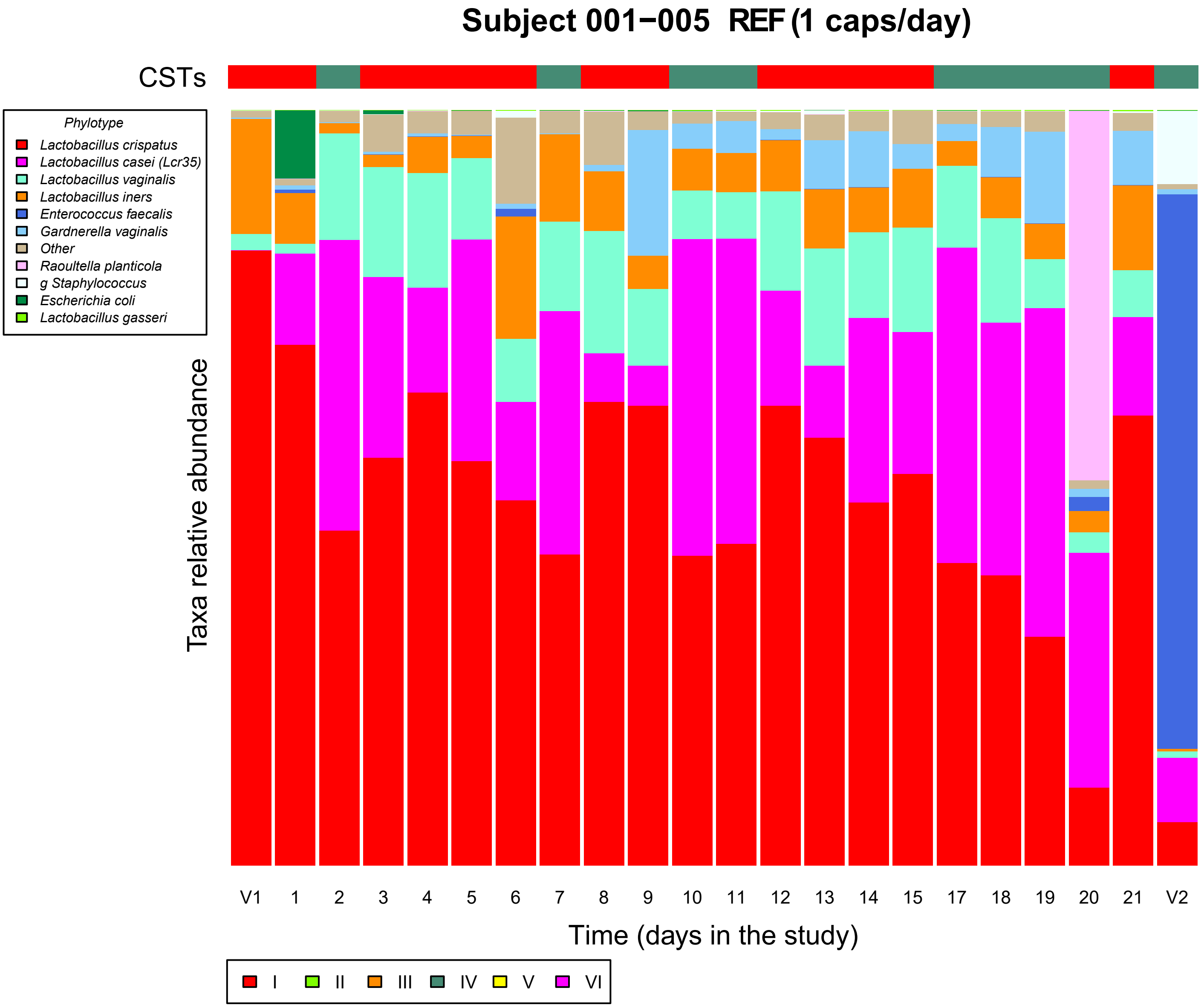

Subject 001−005 REF (1 caps/day)
CSTs
Phylotype
Lactobacillus crispatus
Lactobacillus casei (Lcr35)
Lactobacillus vaginalis
Lactobacillus iners
Enterococcus faecalis
Gardnerella vaginalis
Other
Raoultella planticola
g Staphylococcus
Escherichia coli
Lactobacillus gasseri
Taxa relative abundance
V1
1
2
3
4
5
6
7
8
9
10
11
12
13
14
15
17
18
19
20
21
V2
Time (days in the study)
I
II
III
IV
V
VI

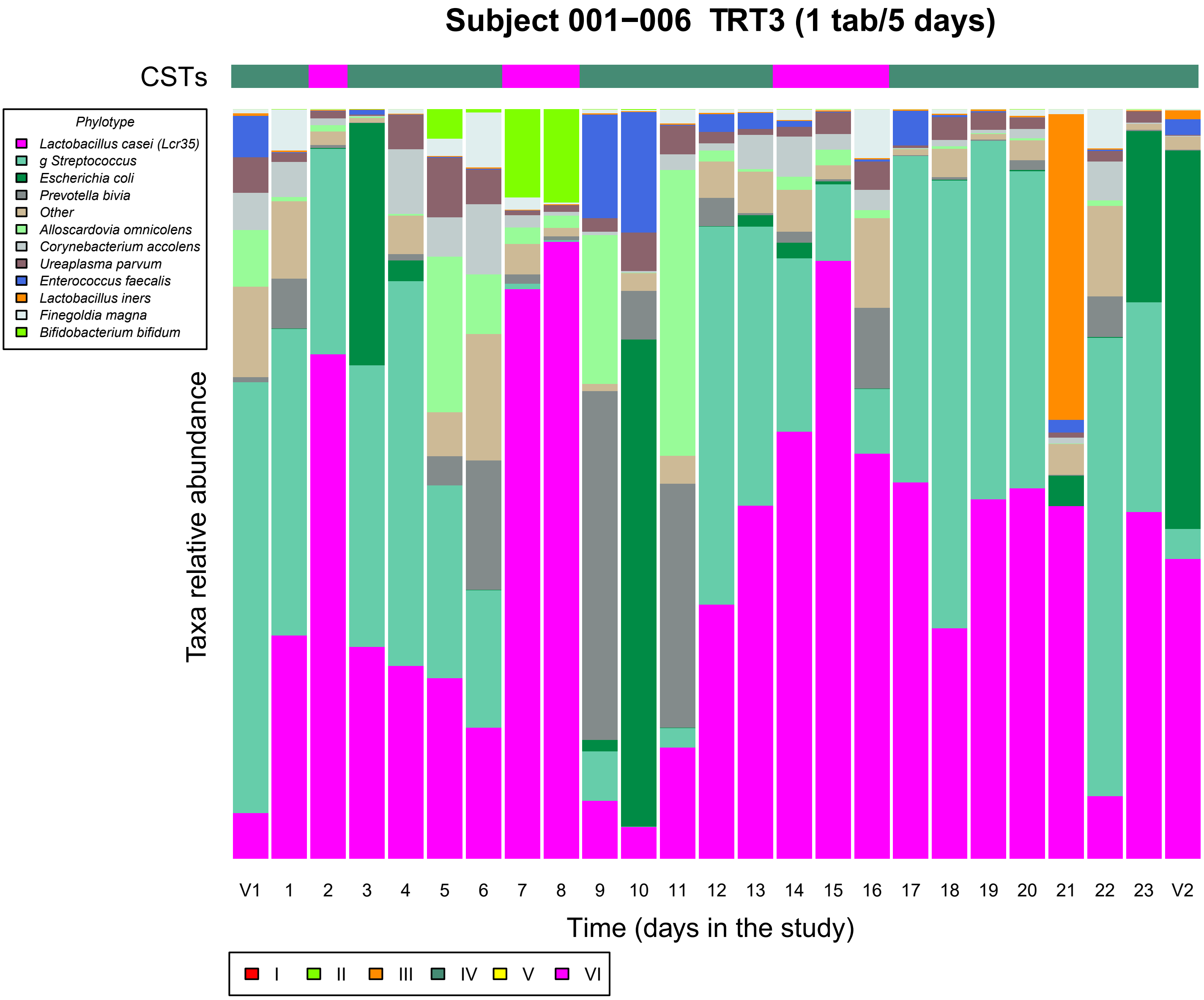

Subject 001−006 TRT3 (1 tab/5 days)
CSTs
Phylotype
Lactobacillus casei (Lcr35)
g Streptococcus
Escherichia coli
Prevotella bivia
Other
Alloscardovia omnicolens
Corynebacterium accolens
Ureaplasma parvum
Enterococcus faecalis
Lactobacillus iners
Finegoldia magna
Bifidobacterium bifidum
Taxa relative abundance
V1 1 2 3 4 5 6 7 8 9 10 11 12 13 14 15 16 17 18 19 20 21 22 23 V2
Time (days in the study)
I II III IV V VI

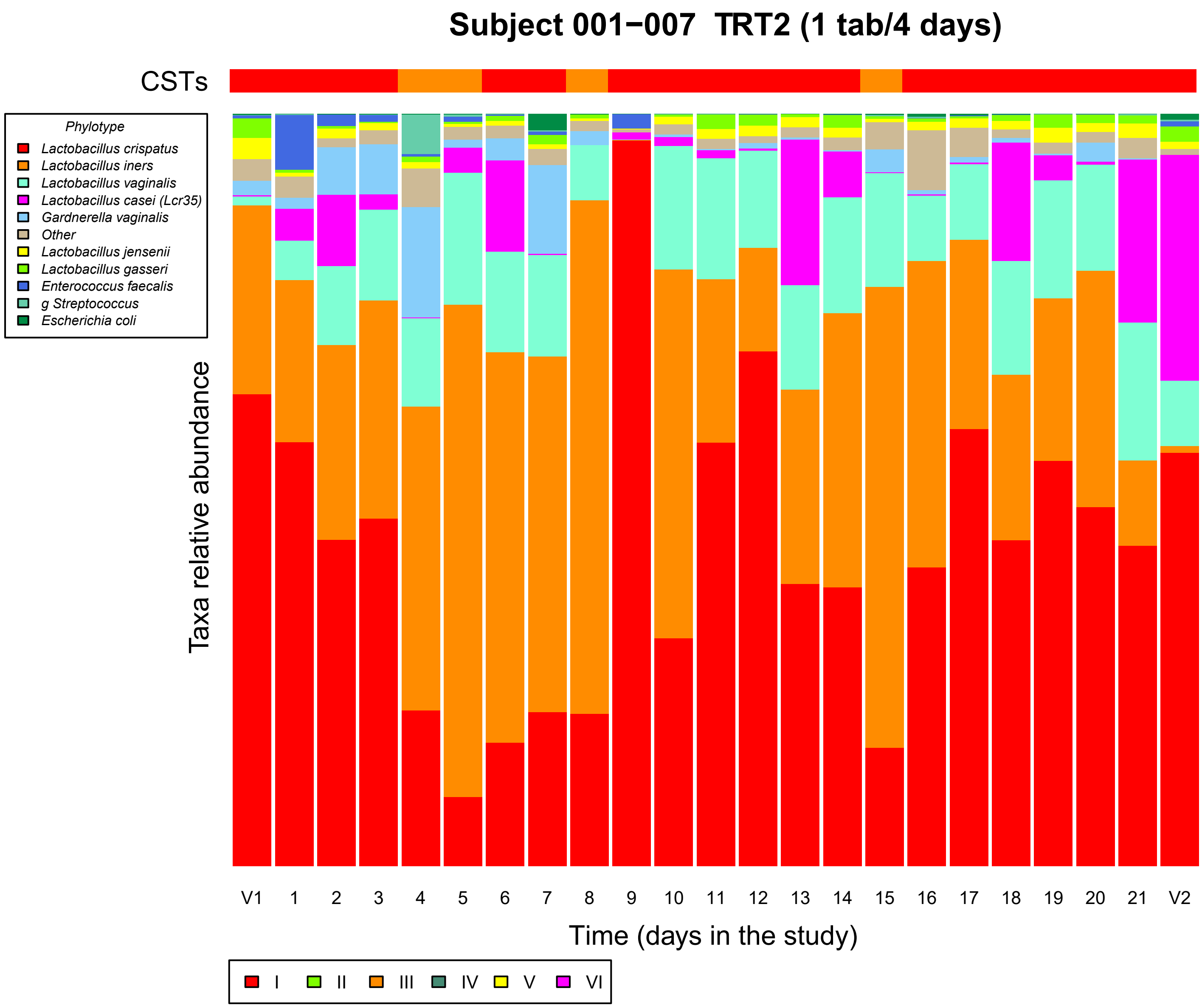
Subject 001−007 TRT2 (1 tab/4 days)
CSTs
Phylotype
Lactobacillus crispatus
Lactobacillus iners
Lactobacillus vaginalis
Lactobacillus casei (Lcr35)
Gardnerella vaginalis
Other
Lactobacillus jensenii
Lactobacillus gasseri
Enterococcus faecalis
g Streptococcus
Escherichia coli
Taxa relative abundance
V1
1
2
3
4
5
6
7
8
9
10
11
12
13
14
15
16
17
18
19
20
21
V2
Time (days in the study)
I
II
III
IV
V
VI

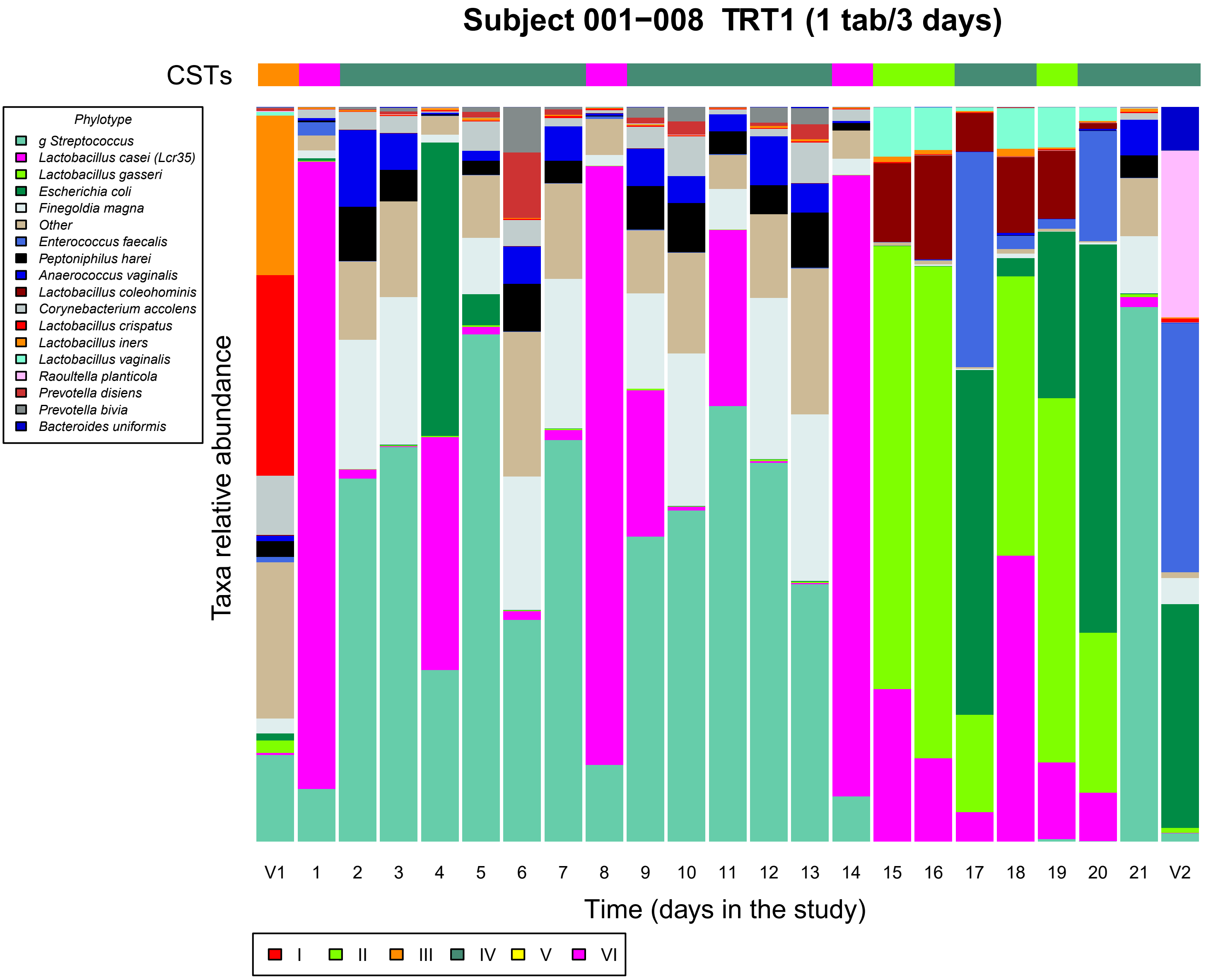

Subject 001−008 TRT1 (1 tab/3 days)
CSTs
Phylotype
g Streptococcus
Lactobacillus casei (Lcr35)
Lactobacillus gasseri
Escherichia coli
Finegoldia magna
Other
Enterococcus faecalis
Peptoniphilus harei
Anaerococcus vaginalis
Lactobacillus coleohominis
Corynebacterium accolens
Lactobacillus crispatus
Lactobacillus iners
Lactobacillus vaginalis
Raoultella planticola
Prevotella disiens
Prevotella bivia
Bacteroides uniformis
Taxa relative abundance
V1 1 2 3 4 5 6 7 8 9 10 11 12 13 14 15 16 17 18 19 20 21 V2
Time (days in the study)
I II III IV V VI

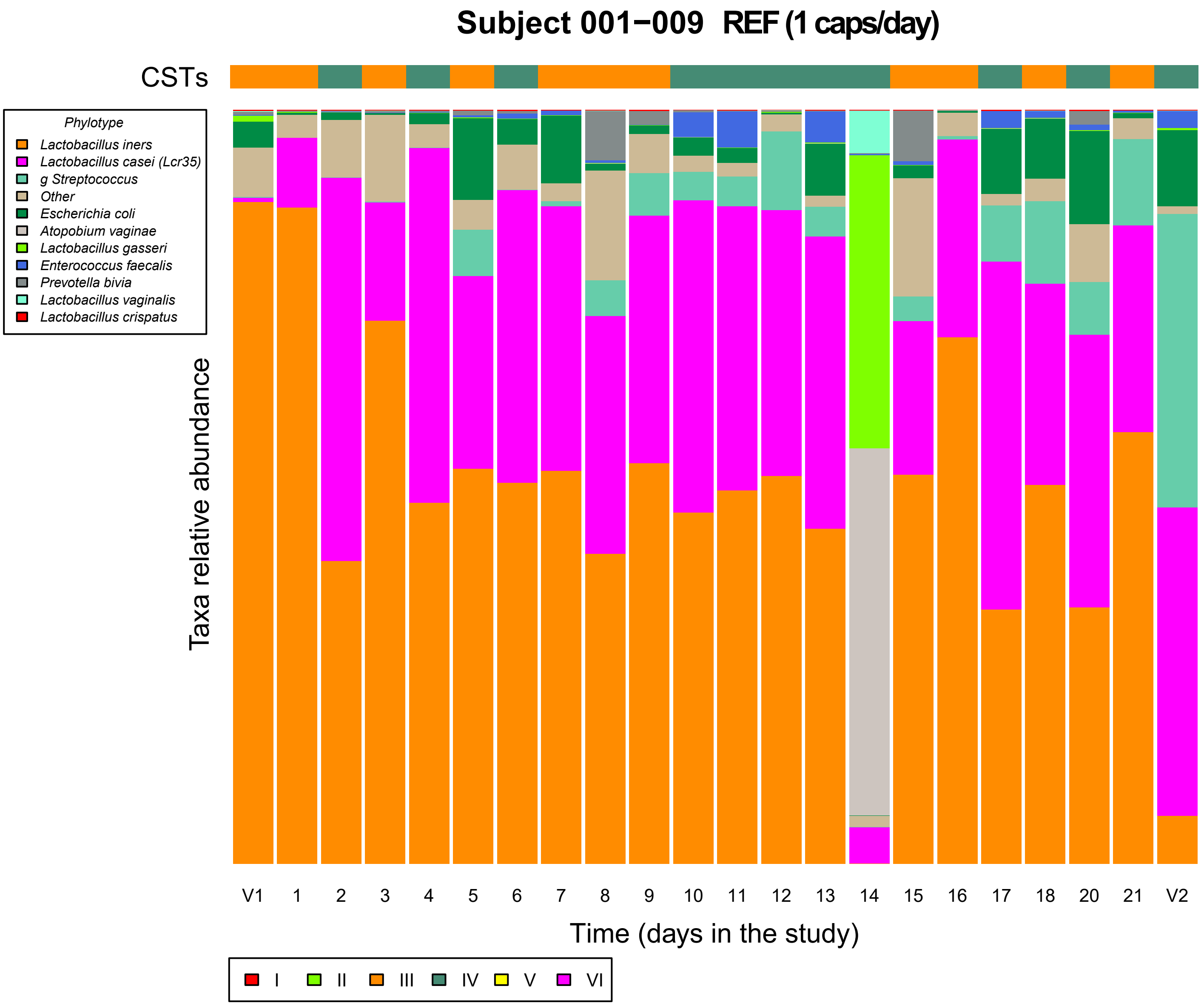
Subject 001−009 REF (1 caps/day)
CSTs
Phylotype
Lactobacillus iners
Lactobacillus casei (Lcr35)
g Streptococcus
Other
Escherichia coli
Atopobium vaginae
Lactobacillus gasseri
Enterococcus faecalis
Prevotella bivia
Lactobacillus vaginalis
Lactobacillus crispatus
Taxa relative abundance
V1
1
2
3
4
5
6
7
8
9
10
11
12
13
14
15
16
17
18
20
21
V2
Time (days in the study)
I
II
III
IV
V
VI

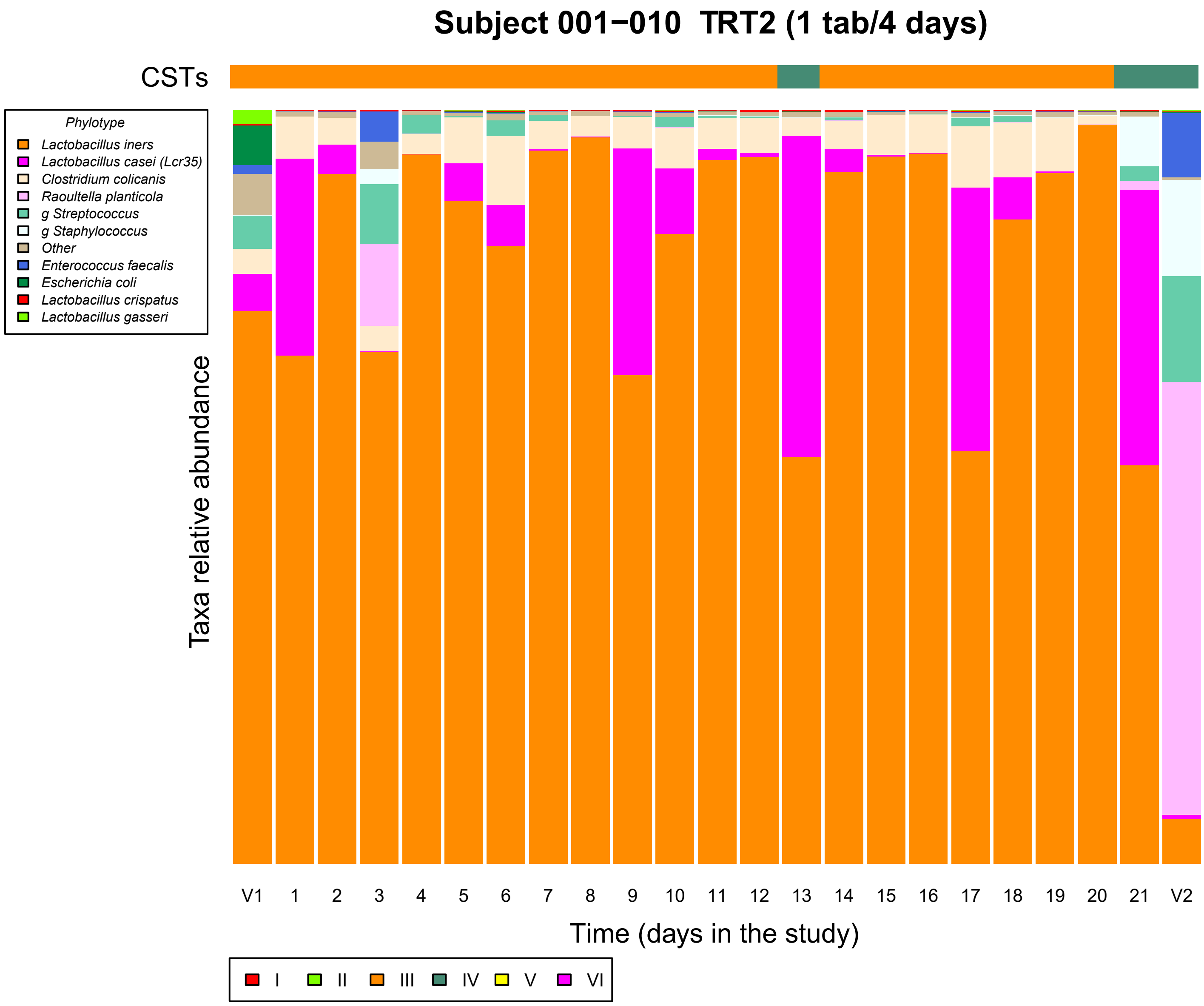

Subject 001−010 TRT2 (1 tab/4 days)
CSTs
Phylotype
Lactobacillus iners
Lactobacillus casei (Lcr35)
Clostridium colicanis
Raoultella planticola
g Streptococcus
g Staphylococcus
Other
Enterococcus faecalis
Escherichia coli
Lactobacillus crispatus
Lactobacillus gasseri
Taxa relative abundance
V1 1 2 3 4 5 6 7 8 9 10 11 12 13 14 15 16 17 18 19 20 21 V2
Time (days in the study)
I II III IV V VI

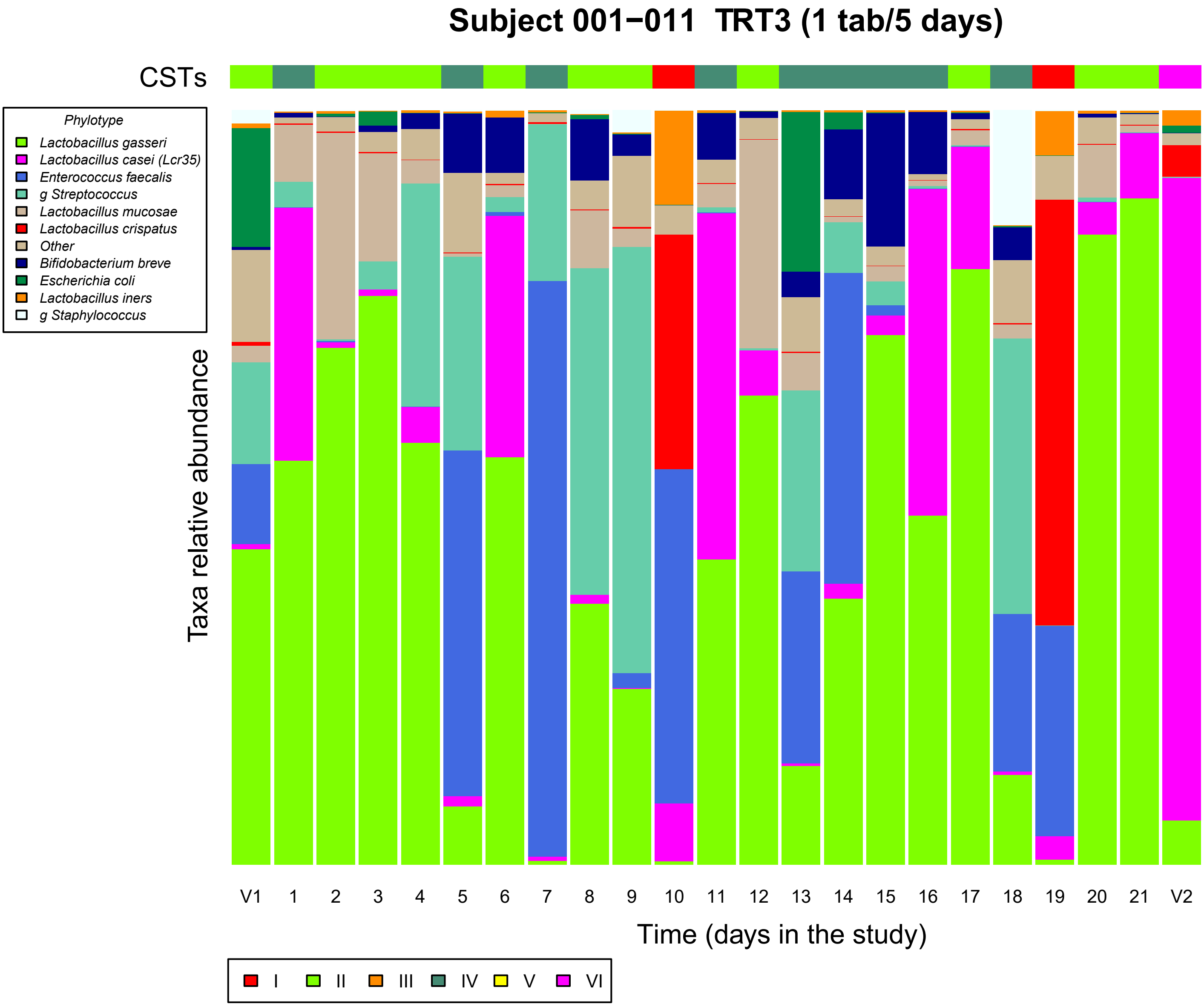
Subject 001−011 TRT3 (1 tab/5 days)
CSTs
Phylotype
Lactobacillus gasseri
Lactobacillus casei (Lcr35)
Enterococcus faecalis
g Streptococcus
Lactobacillus mucosae
Lactobacillus crispatus
Other
Bifidobacterium breve
Escherichia coli
Lactobacillus iners
g Staphylococcus
Taxa relative abundance
V1
1
2
3
4
5
6
7
8
9
10
11
12
13
14
15
16
17
18
19
20
21
V2
Time (days in the study)
I
II
III
IV
V
VI

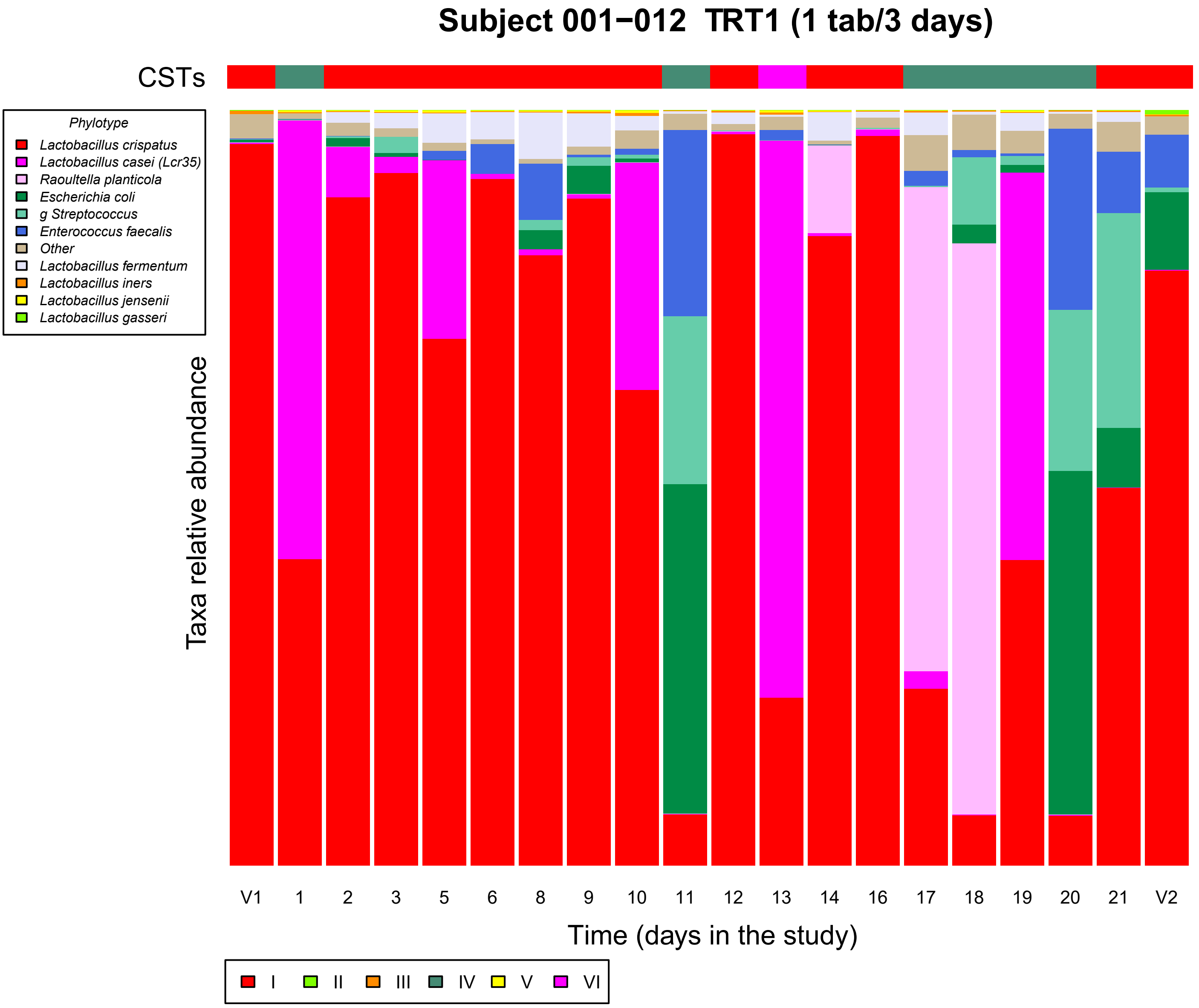

Subject 001−012 TRT1 (1 tab/3 days)
CSTs
Phylotype
Lactobacillus crispatus
Lactobacillus casei (Lcr35)
Raoultella planticola
Escherichia coli
g Streptococcus
Enterococcus faecalis
Other
Lactobacillus fermentum
Lactobacillus iners
Lactobacillus jensenii
Lactobacillus gasseri
Taxa relative abundance
V1
1
2
3
5
6
8
9
10
11
12
13
14
16
17
18
19
20
21
V2
Time (days in the study)
I
II
III
IV
V
VI

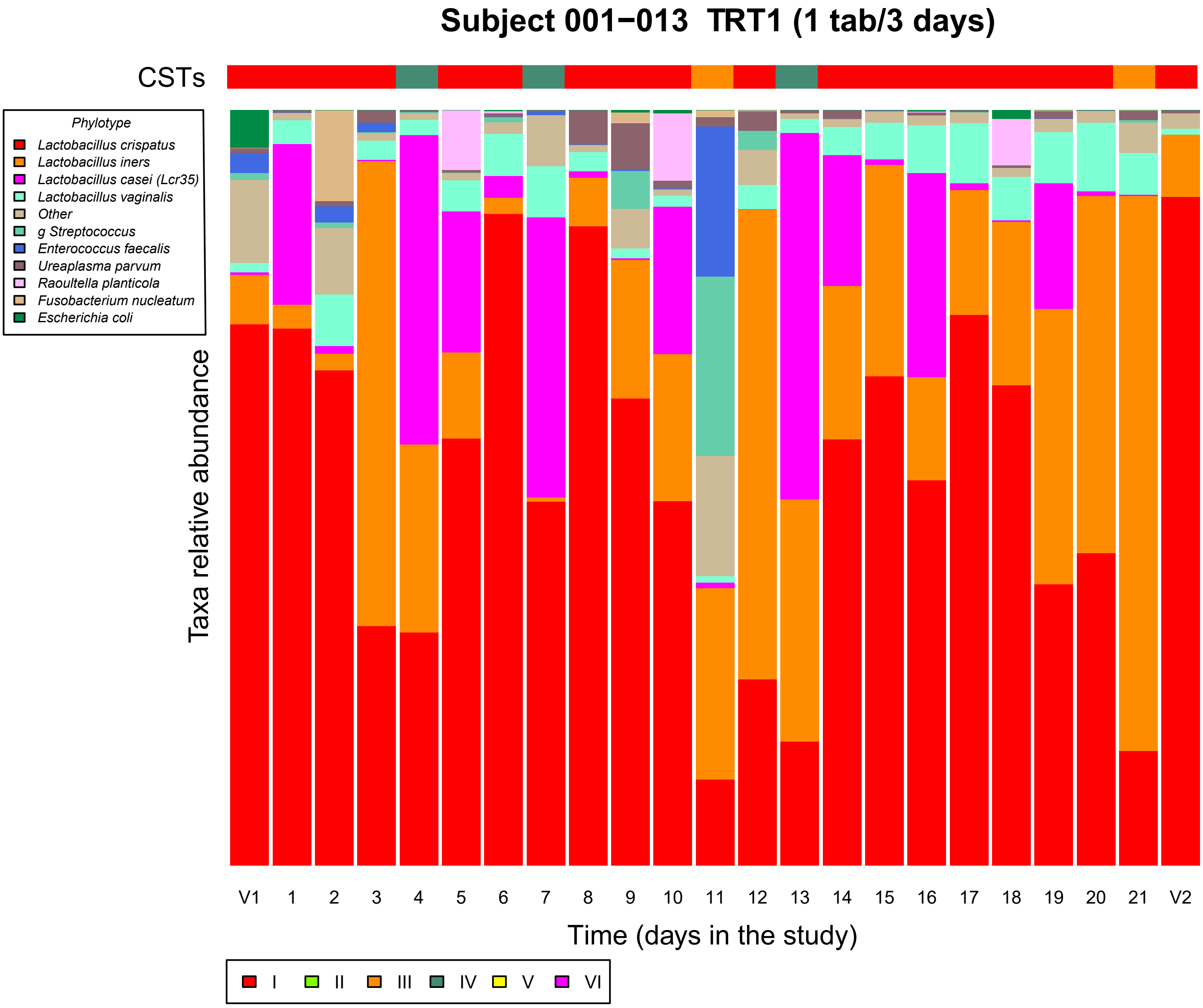
Subject 001−013 TRT1 (1 tab/3 days)
CSTs
Phylotype
Lactobacillus crispatus
Lactobacillus iners
Lactobacillus casei (Lcr35)
Lactobacillus vaginalis
Other
g Streptococcus
Enterococcus faecalis
Ureaplasma parvum
Raoultella planticola
Fusobacterium nucleatum
Escherichia coli
Taxa relative abundance
V1
1
2
3
4
5
6
7
8
9
10
11
12
13
14
15
16
17
18
19
20
21
V2
Time (days in the study)
I
II
III
IV
V
VI

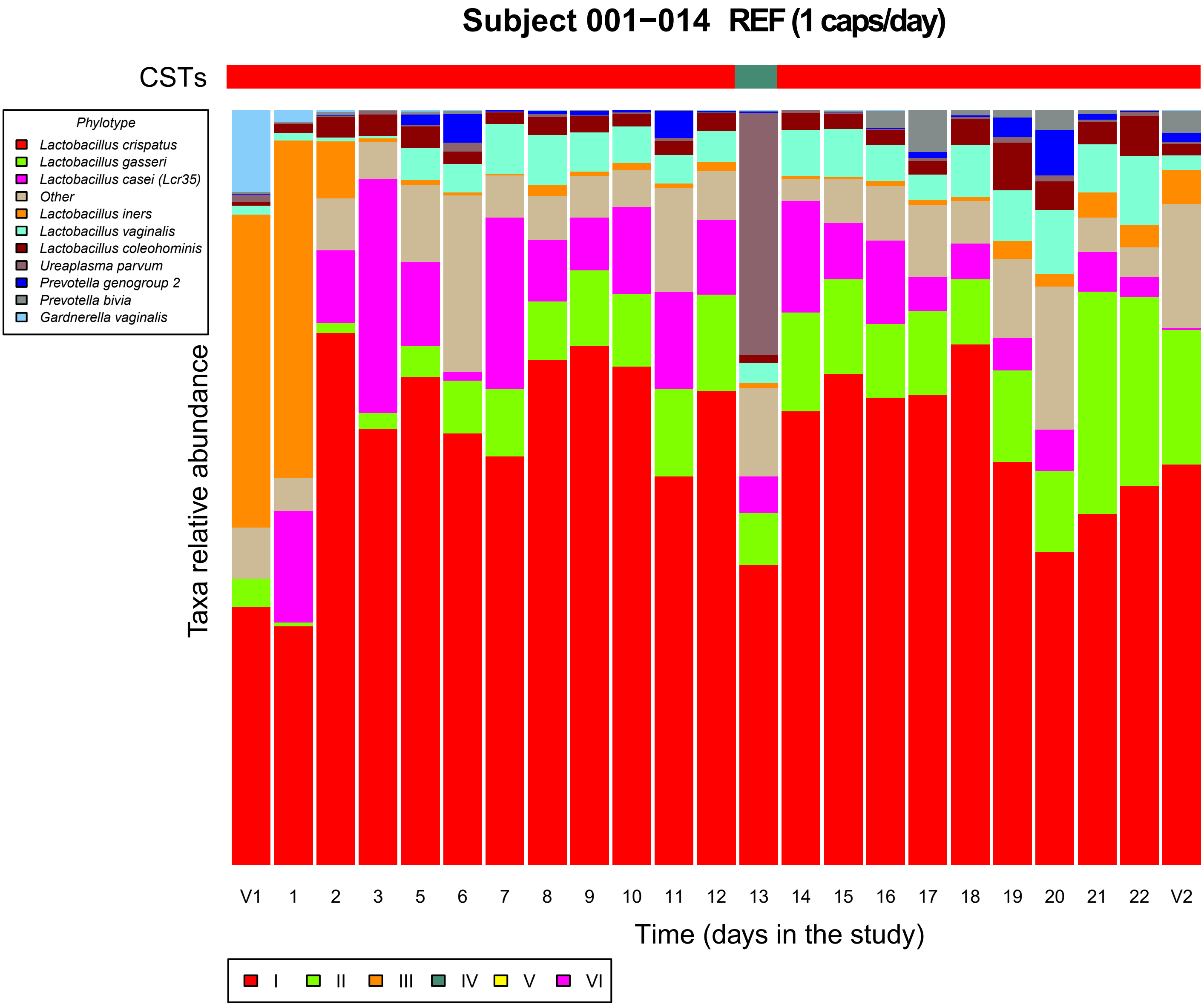

Subject 001−014 REF (1 caps/day)
CSTs
Phylotype
Lactobacillus crispatus
Lactobacillus gasseri
Lactobacillus casei (Lcr35)
Other
Lactobacillus iners
Lactobacillus vaginalis
Lactobacillus coleohominis
Ureaplasma parvum
Prevotella genogroup 2
Prevotella bivia
Gardnerella vaginalis
Taxa relative abundance
V1 1 2 3 5 6 7 8 9 10 11 12 13 14 15 16 17 18 19 20 21 22 V2
Time (days in the study)
I II III IV V VI

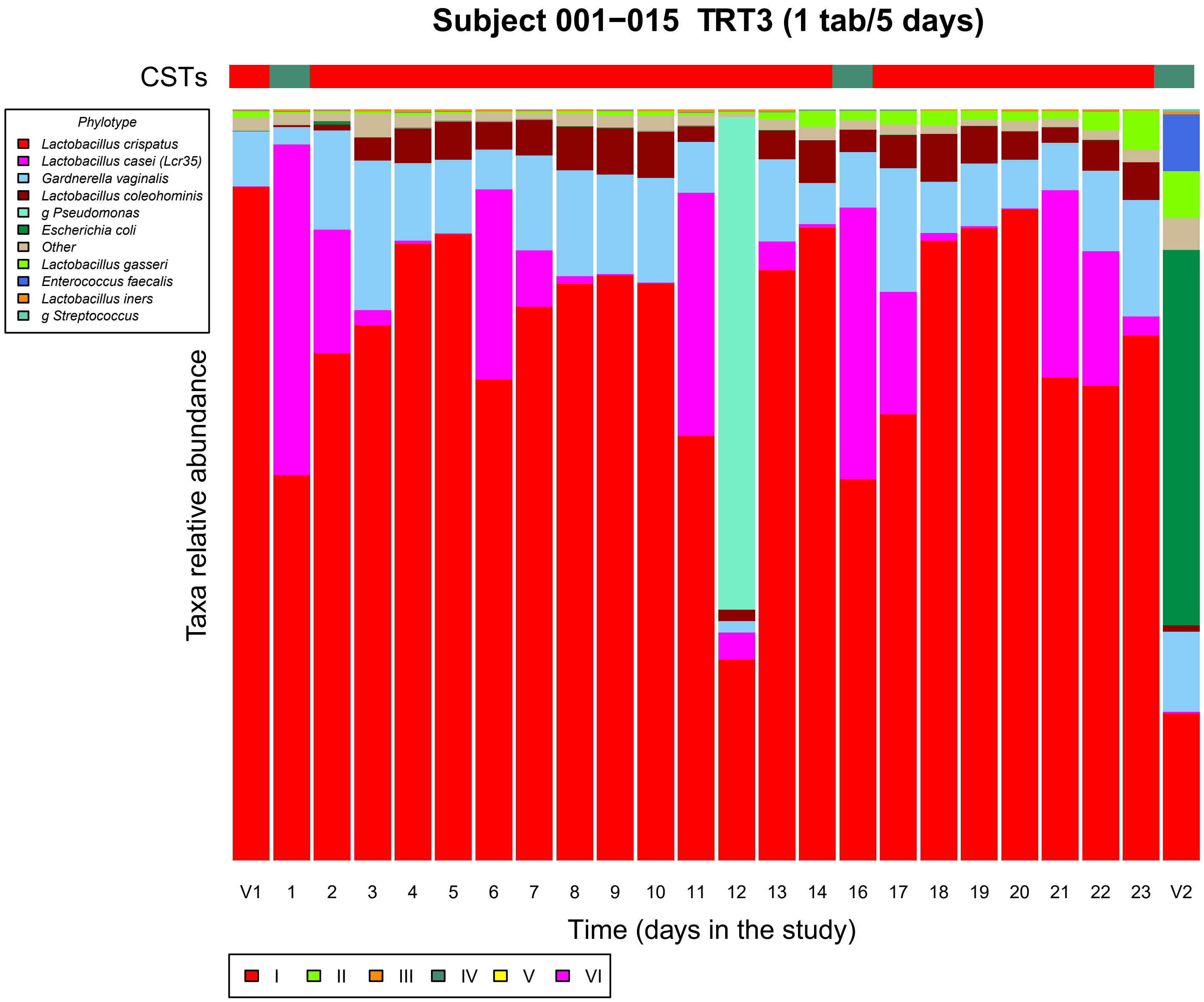

Subject 001−015 TRT3 (1 tab/5 days)
CSTs
Phylotype
Lactobacillus crispatus
Lactobacillus casei (Lcr35)
Gardnerella vaginalis
Lactobacillus coleohominis
g Pseudomonas
Escherichia coli
Other
Lactobacillus gasseri
Enterococcus faecalis
Lactobacillus iners
g Streptococcus
Taxa relative abundance
V1 1 2 3 4 5 6 7 8 9 10 11 12 13 14 16 17 18 19 20 21 22 23 V2
Time (days in the study)
I II III IV V VI

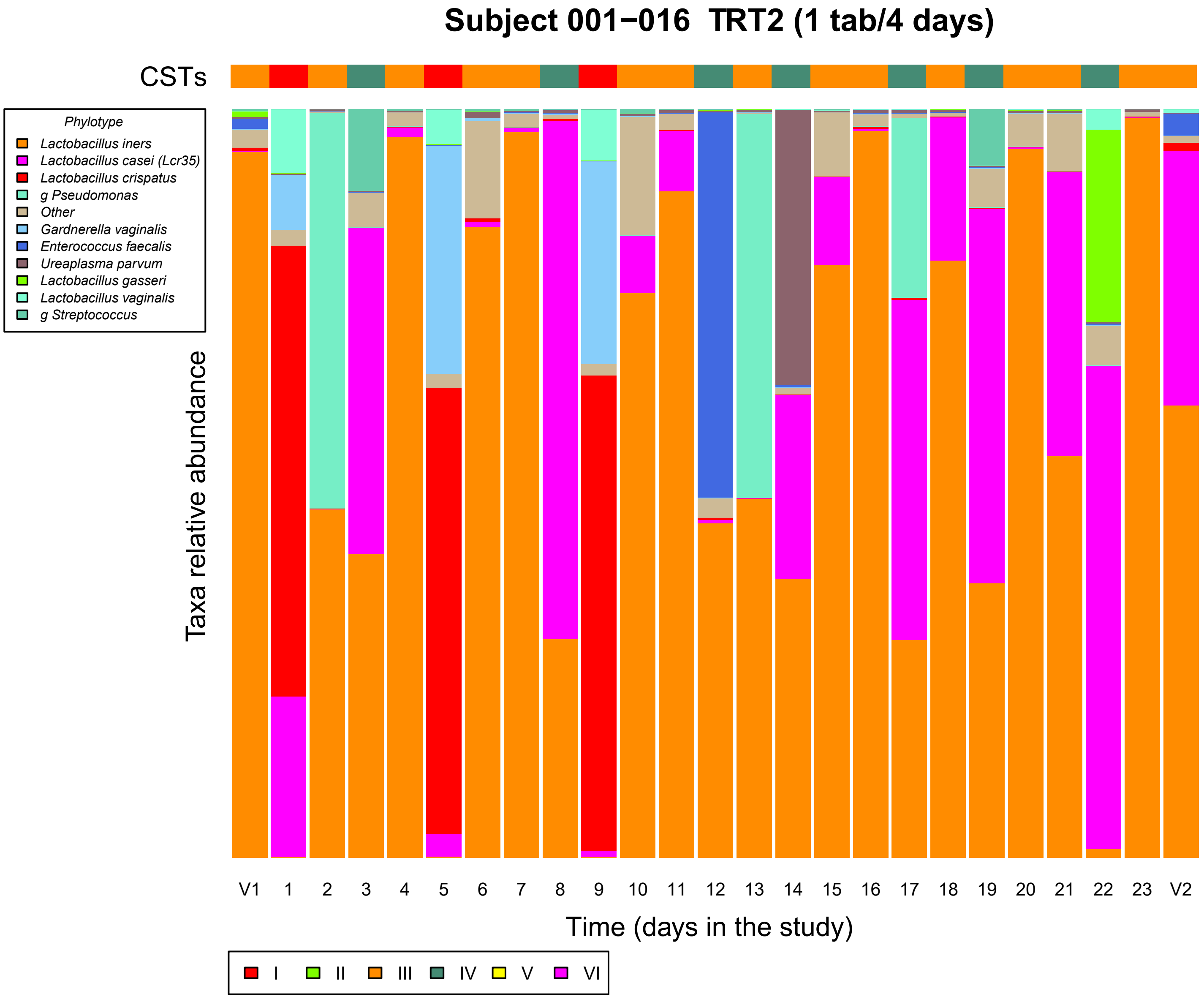

Subject 001−016 TRT2 (1 tab/4 days)
CSTs
Phylotype
Lactobacillus iners
Lactobacillus casei (Lcr35)
Lactobacillus crispatus
g Pseudomonas
Other
Gardnerella vaginalis
Enterococcus faecalis
Ureaplasma parvum
Lactobacillus gasseri
Lactobacillus vaginalis
g Streptococcus
Taxa relative abundance
V1
1
2
3
4
5
6
7
8
9
10
11
12
13
14
15
16
17
18
19
20
21
22
23
V2
Time (days in the study)
I
II
III
IV
V
VI

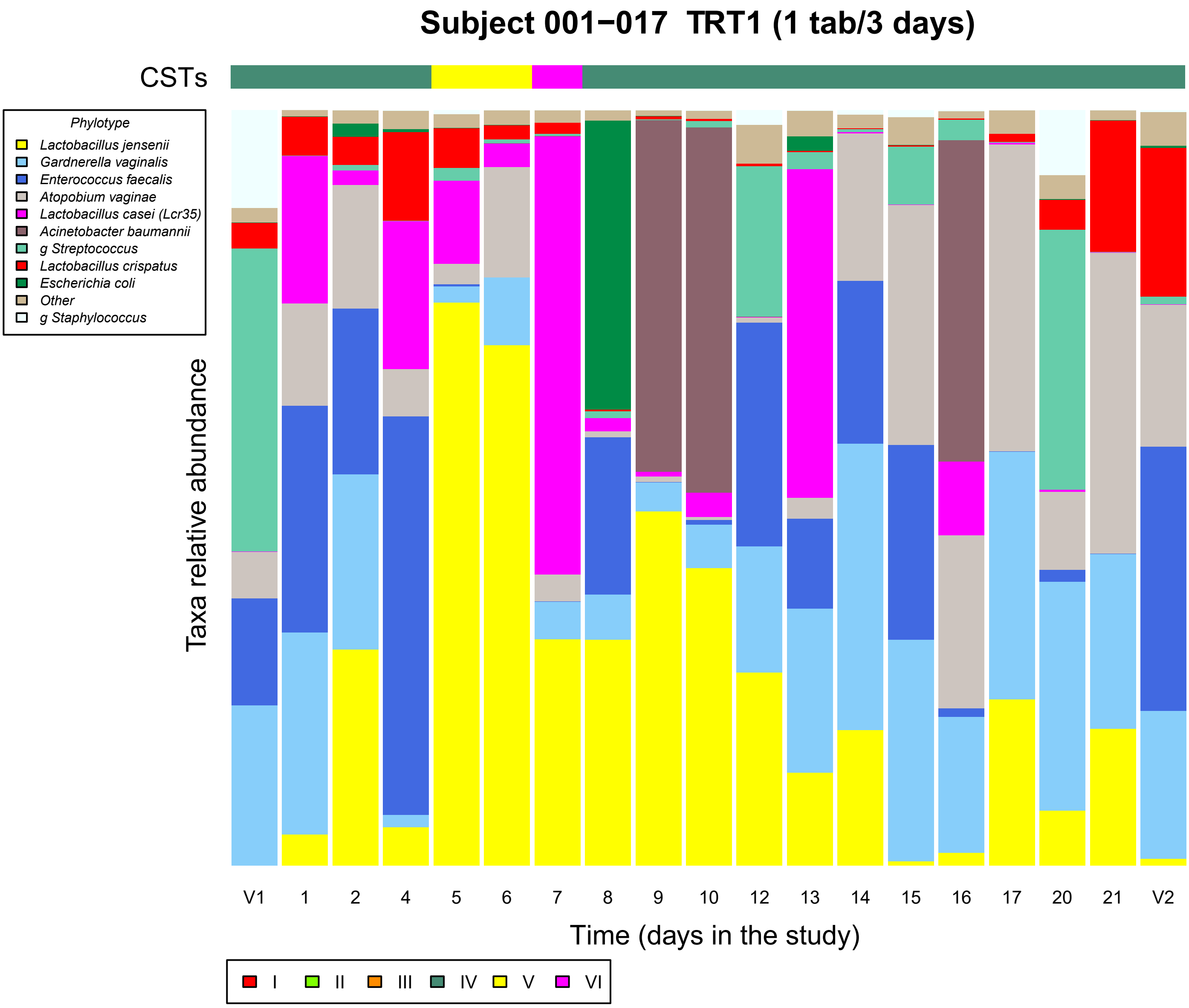

Subject 001−017 TRT1 (1 tab/3 days)
CSTs
Phylotype
Lactobacillus jensenii
Gardnerella vaginalis
Enterococcus faecalis
Atopobium vaginae
Lactobacillus casei (Lcr35)
Acinetobacter baumannii
g Streptococcus
Lactobacillus crispatus
Escherichia coli
Other
g Staphylococcus
Taxa relative abundance
V1
1
2
4
5
6
7
8
9
10
12
13
14
15
16
17
20
21
V2
Time (days in the study)
I
II
III
IV
V
VI

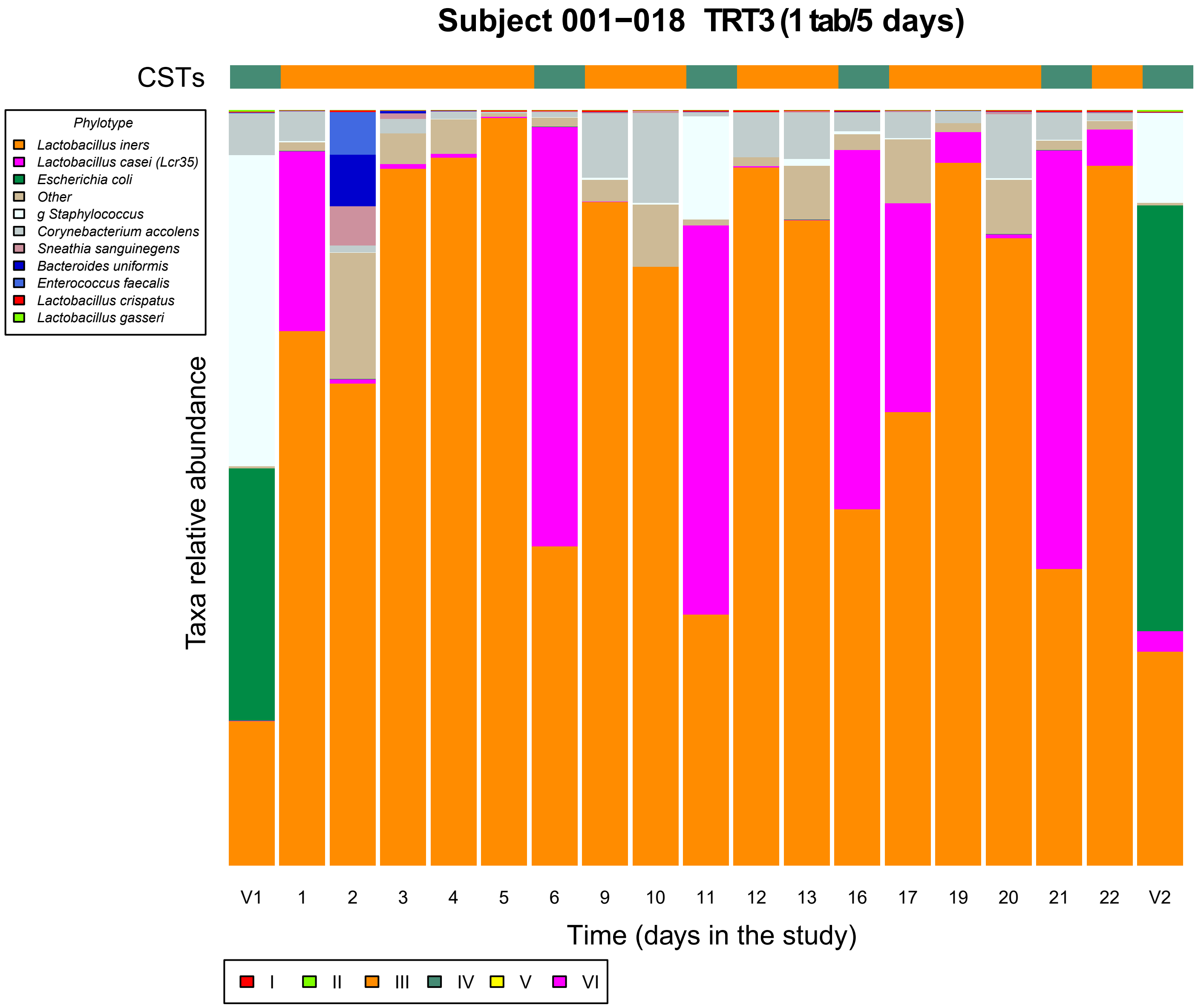
Subject 001−018 TRT3 (1 tab/5 days)
CSTs
Phylotype
Lactobacillus iners
Lactobacillus casei (Lcr35)
Escherichia coli
Other
g Staphylococcus
Corynebacterium accolens
Sneathia sanguinegens
Bacteroides uniformis
Enterococcus faecalis
Lactobacillus crispatus
Lactobacillus gasseri
Taxa relative abundance
V1
1
2
3
4
5
6
9
10
11
12
13
16
17
19
20
21
22
V2
Time (days in the study)
I
II
III
IV
V
VI

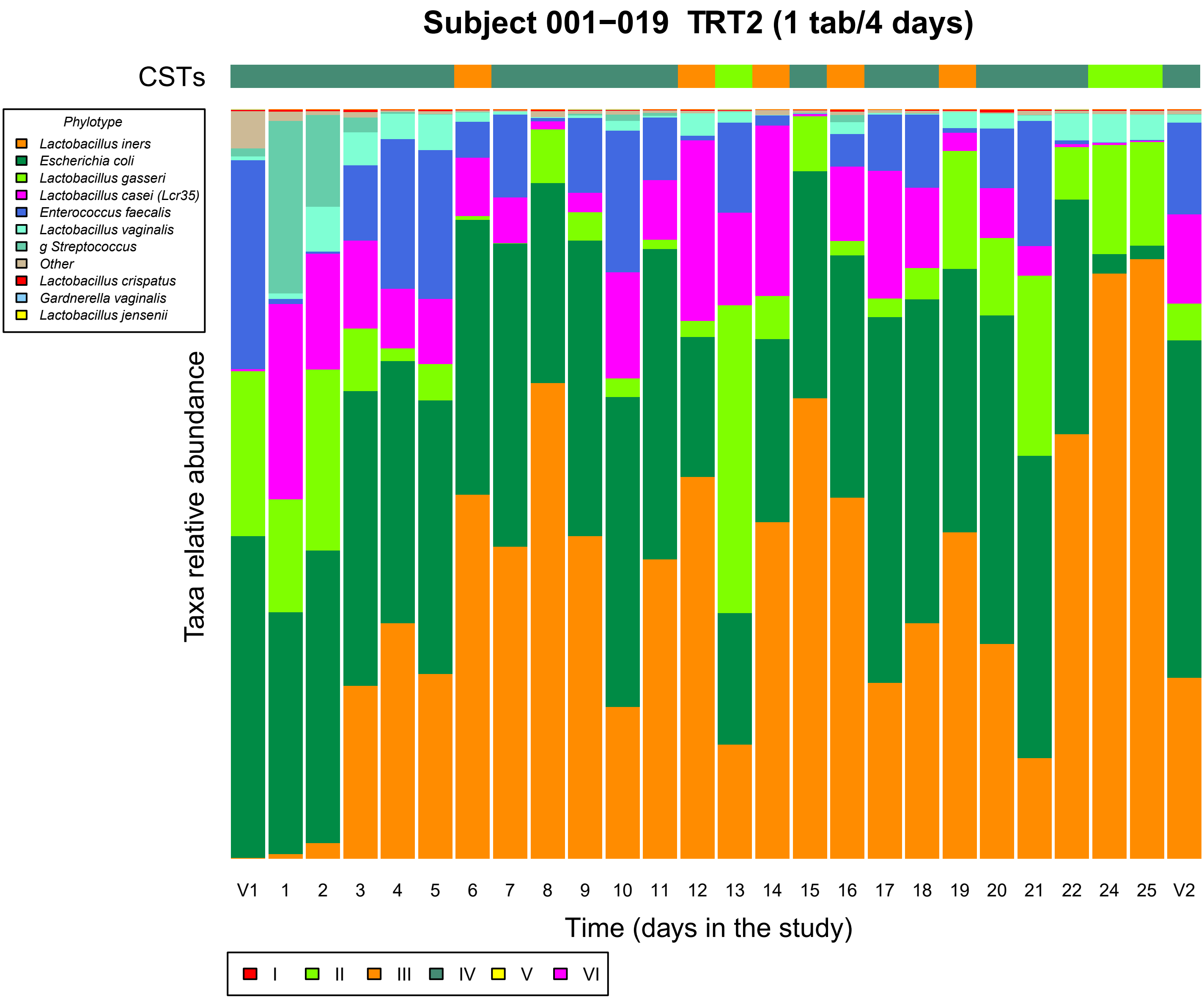
Subject 001−019 TRT2 (1 tab/4 days)
CSTs
Phylotype
Lactobacillus iners
Escherichia coli
Lactobacillus gasseri
Lactobacillus casei (Lcr35)
Enterococcus faecalis
Lactobacillus vaginalis
g Streptococcus
Other
Lactobacillus crispatus
Gardnerella vaginalis
Lactobacillus jensenii
Taxa relative abundance
V1
1
2
3
4
5
6
7
8
9
10
11
12
13
14
15
16
17
18
19
20
21
22
24
25
V2
Time (days in the study)
I
II
III
IV
V
VI

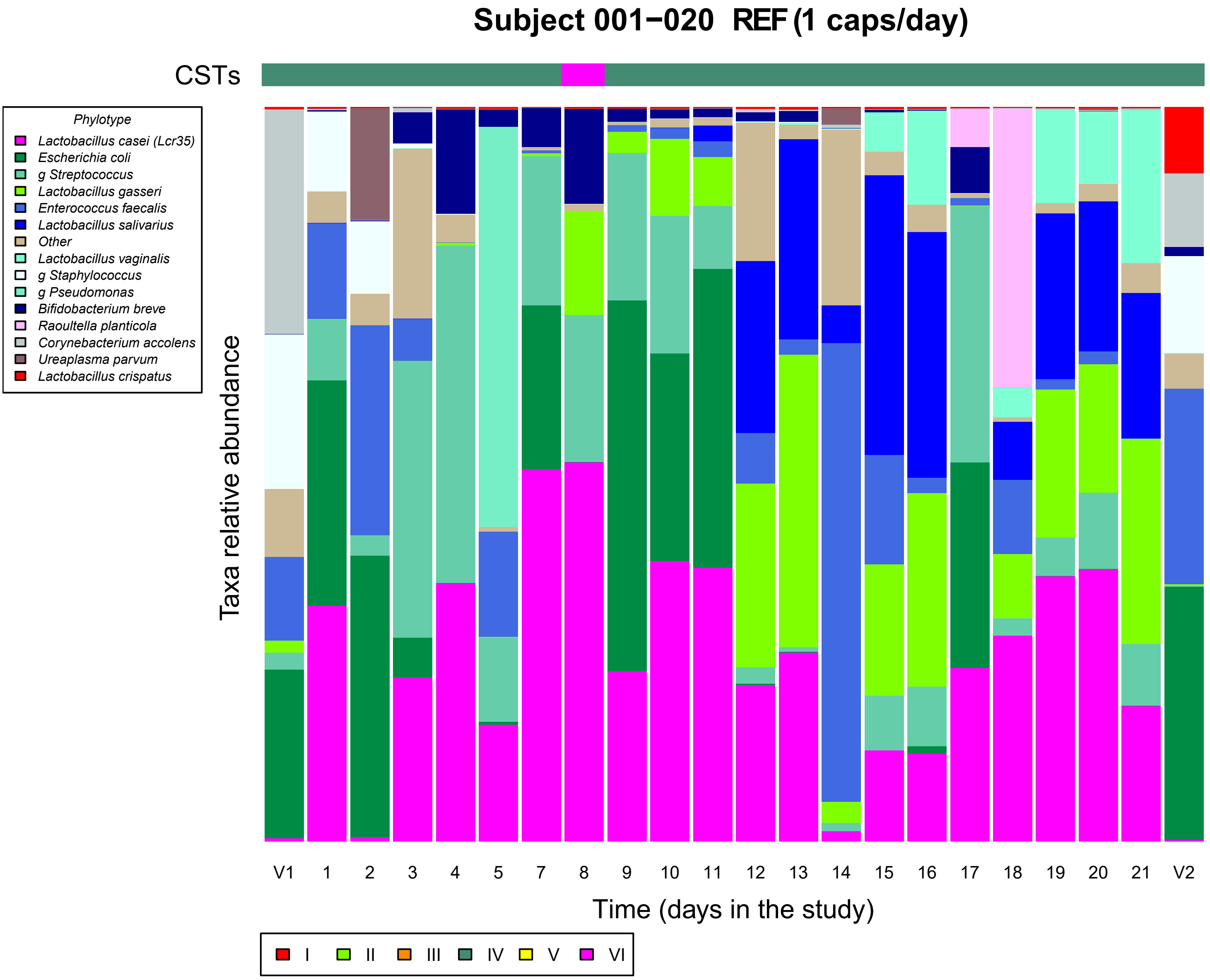

Subject 001−020 REF (1 caps/day)
CSTs
Phylotype
Lactobacillus casei (Lcr35)
Escherichia coli
g Streptococcus
Lactobacillus gasseri
Enterococcus faecalis
Lactobacillus salivarius
Other
Lactobacillus vaginalis
g Staphylococcus
g Pseudomonas
Bifidobacterium breve
Raoultella planticola
Corynebacterium accolens
Ureaplasma parvum
Lactobacillus crispatus
Taxa relative abundance
V1 1 2 3 4 5 7 8 9 10 11 12 13 14 15 16 17 18 19 20 21 V2
Time (days in the study)
I II III IV V VI

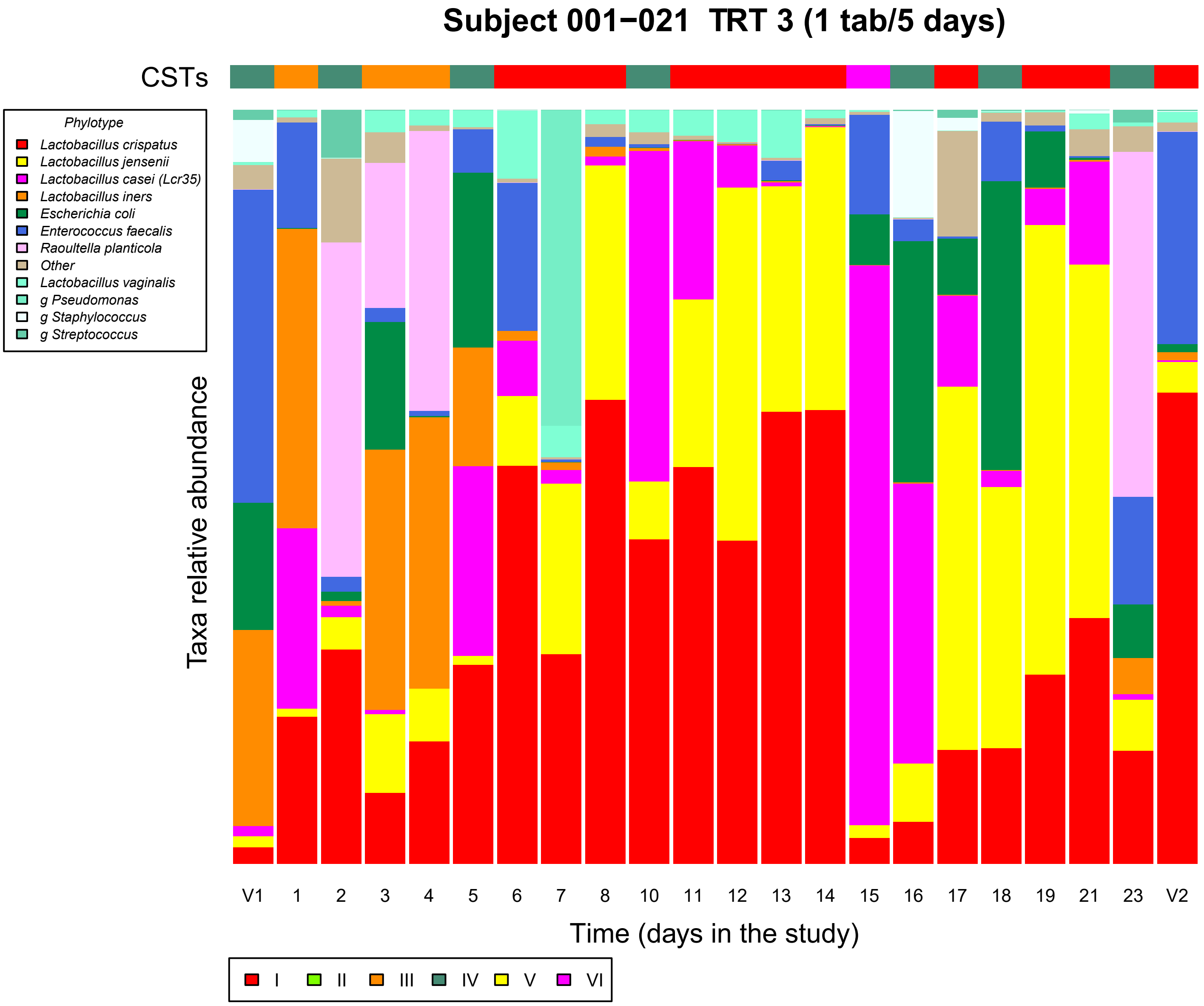
Subject 001−021 TRT 3 (1 tab/5 days)
CSTs
Phylotype
Lactobacillus crispatus
Lactobacillus jensenii
Lactobacillus casei (Lcr35)
Lactobacillus iners
Escherichia coli
Enterococcus faecalis
Raoultella planticola
Other
Lactobacillus vaginalis
g Pseudomonas
g Staphylococcus
g Streptococcus
Taxa relative abundance
V1
1
2
3
4
5
6
7
8
10
11
12
13
14
15
16
17
18
19
21
23
V2
Time (days in the study)
I
II
III
IV
V
VI

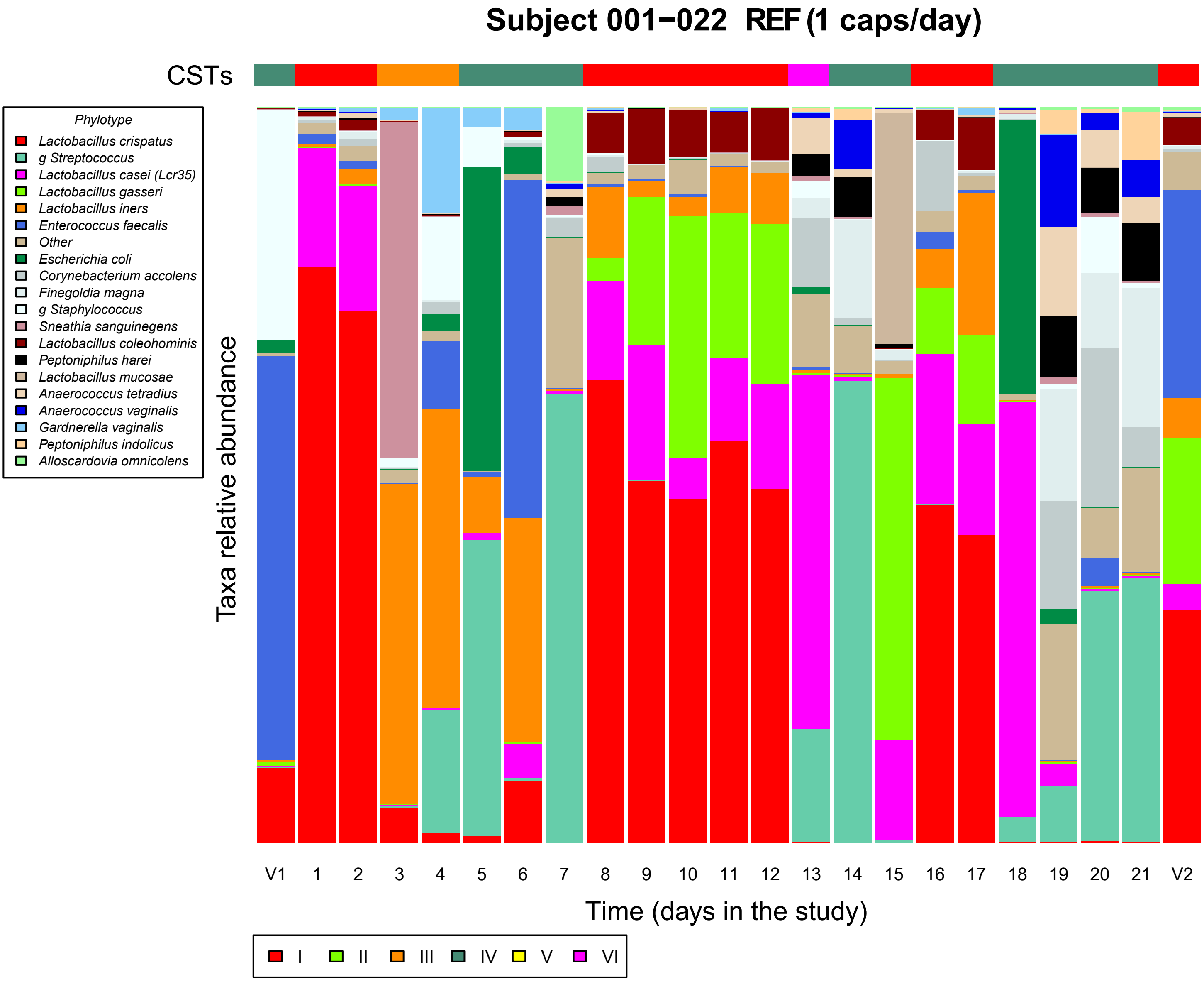
Subject 001−022 REF (1 caps/day)
CSTs
Phylotype
Lactobacillus crispatus
g Streptococcus
Lactobacillus casei (Lcr35)
Lactobacillus gasseri
Lactobacillus iners
Enterococcus faecalis
Other
Escherichia coli
Corynebacterium accolens
Finegoldia magna
g Staphylococcus
Sneathia sanguinegens
Lactobacillus coleohominis
Peptoniphilus harei
Lactobacillus mucosae
Anaerococcus tetradius
Anaerococcus vaginalis
Gardnerella vaginalis
Peptoniphilus indolicus
Alloscardovia omnicolens
Taxa relative abundance
V1 1 2 3 4 5 6 7 8 9 10 11 12 13 14 15 16 17 18 19 20 21 V2
Time (days in the study)
I II III IV V VI

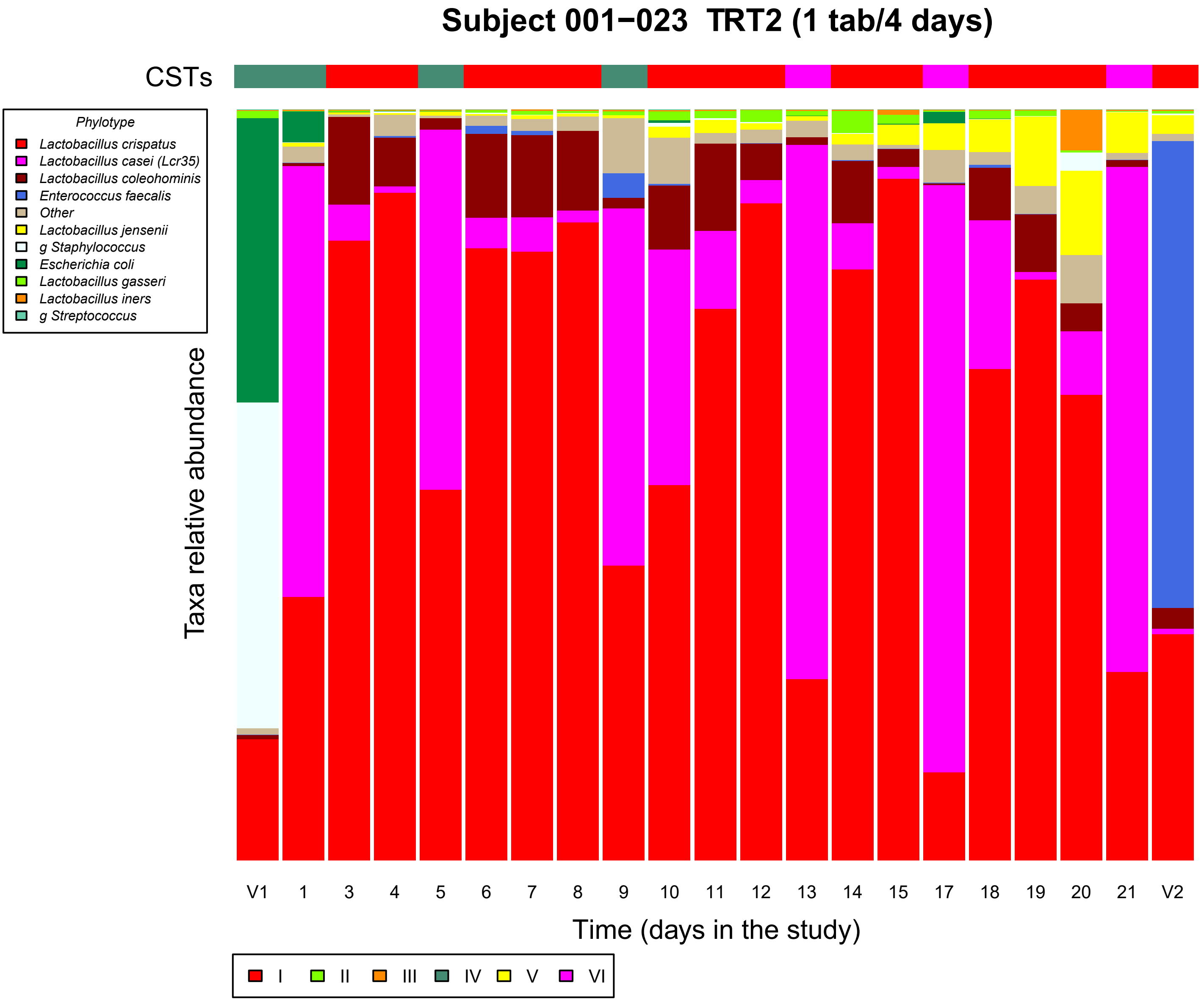

Subject 001−023 TRT2 (1 tab/4 days)
CSTs
Phylotype
Lactobacillus crispatus
Lactobacillus casei (Lcr35)
Lactobacillus coleohominis
Enterococcus faecalis
Other
Lactobacillus jensenii
g Staphylococcus
Escherichia coli
Lactobacillus gasseri
Lactobacillus iners
g Streptococcus
Taxa relative abundance
V1 1 3 4 5 6 7 8 9 10 11 12 13 14 15 17 18 19 20 21 V2
Time (days in the study)
I II III IV V VI

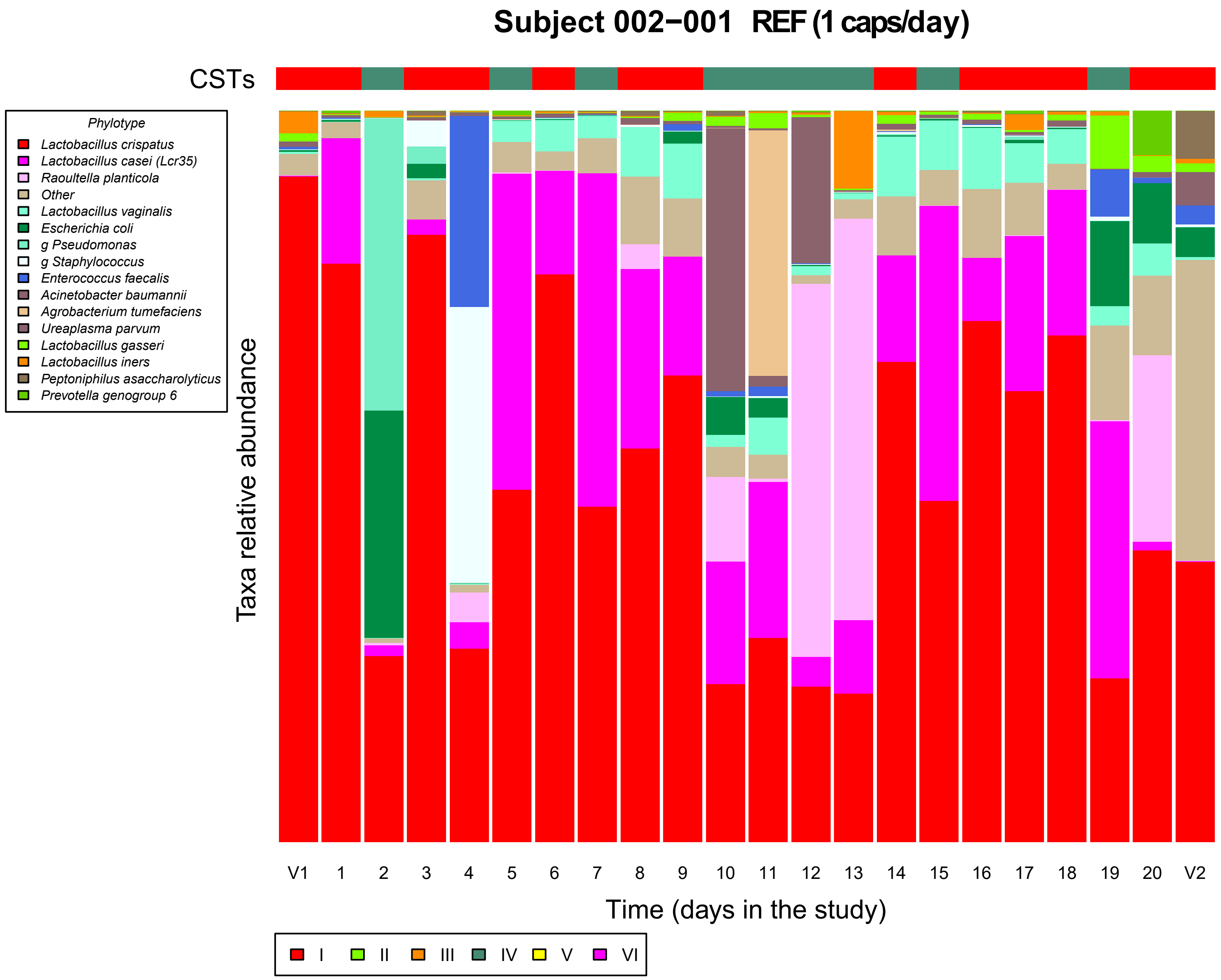
Subject 002−001 REF (1 caps/day)
CSTs
Phylotype
Lactobacillus crispatus
Lactobacillus casei (Lcr35)
Raoultella planticola
Other
Lactobacillus vaginalis
Escherichia coli
g Pseudomonas
g Staphylococcus
Enterococcus faecalis
Acinetobacter baumannii
Agrobacterium tumefaciens
Ureaplasma parvum
Lactobacillus gasseri
Lactobacillus iners
Peptoniphilus asaccharolyticus
Prevotella genogroup 6
Taxa relative abundance
V1 1 2 3 4 5 6 7 8 9 10 11 12 13 14 15 16 17 18 19 20 V2
Time (days in the study)
I II III IV V VI

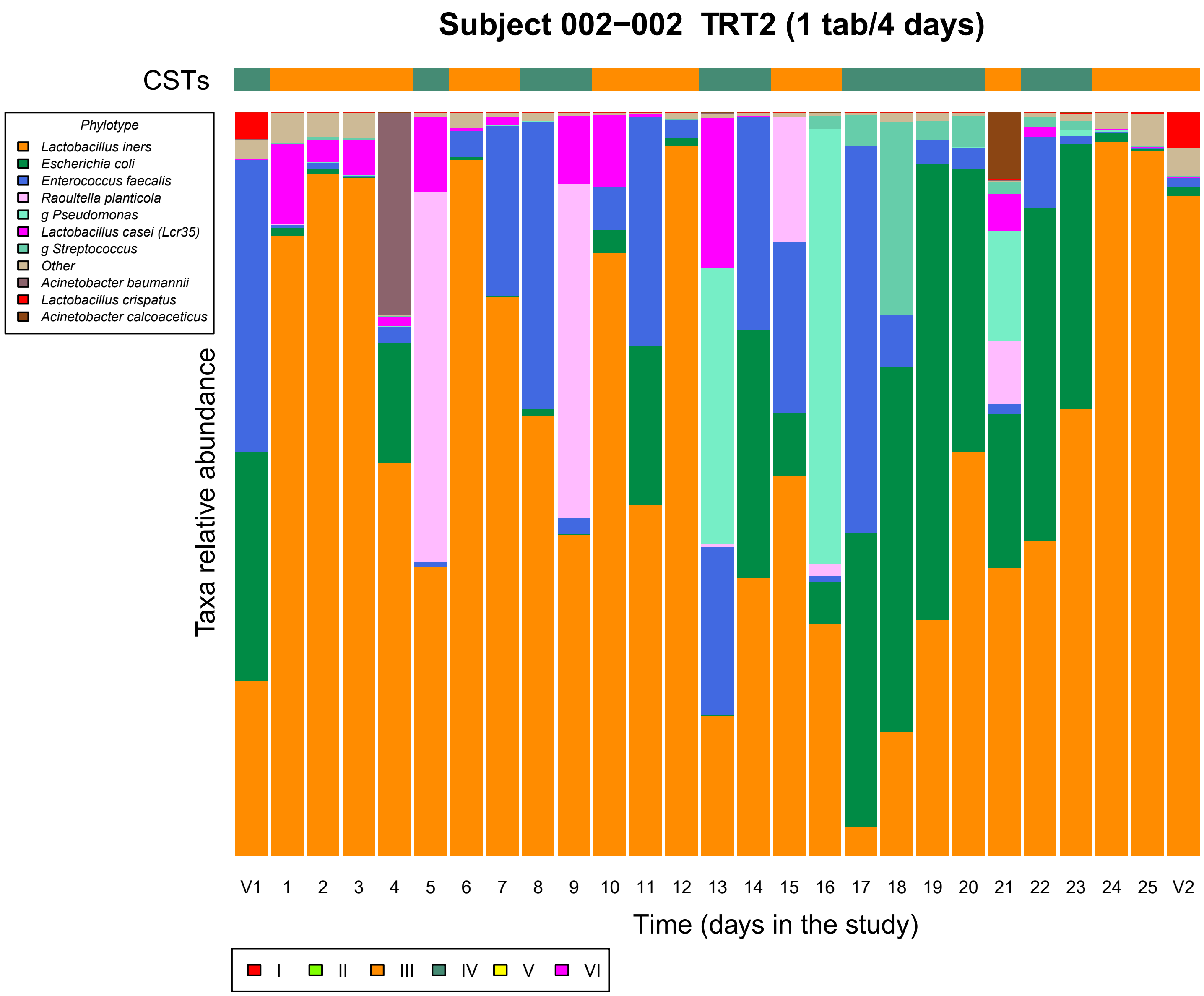
Subject 002−002 TRT2 (1 tab/4 days)
CSTs
Phylotype
Lactobacillus iners
Escherichia coli
Enterococcus faecalis
Raoultella planticola
g Pseudomonas
Lactobacillus casei (Lcr35)
g Streptococcus
Other
Acinetobacter baumannii
Lactobacillus crispatus
Acinetobacter calcoaceticus
Taxa relative abundance
V1 1 2 3 4 5 6 7 8 9 10 11 12 13 14 15 16 17 18 19 20 21 22 23 24 25 V2
Time (days in the study)
I II III IV V VI

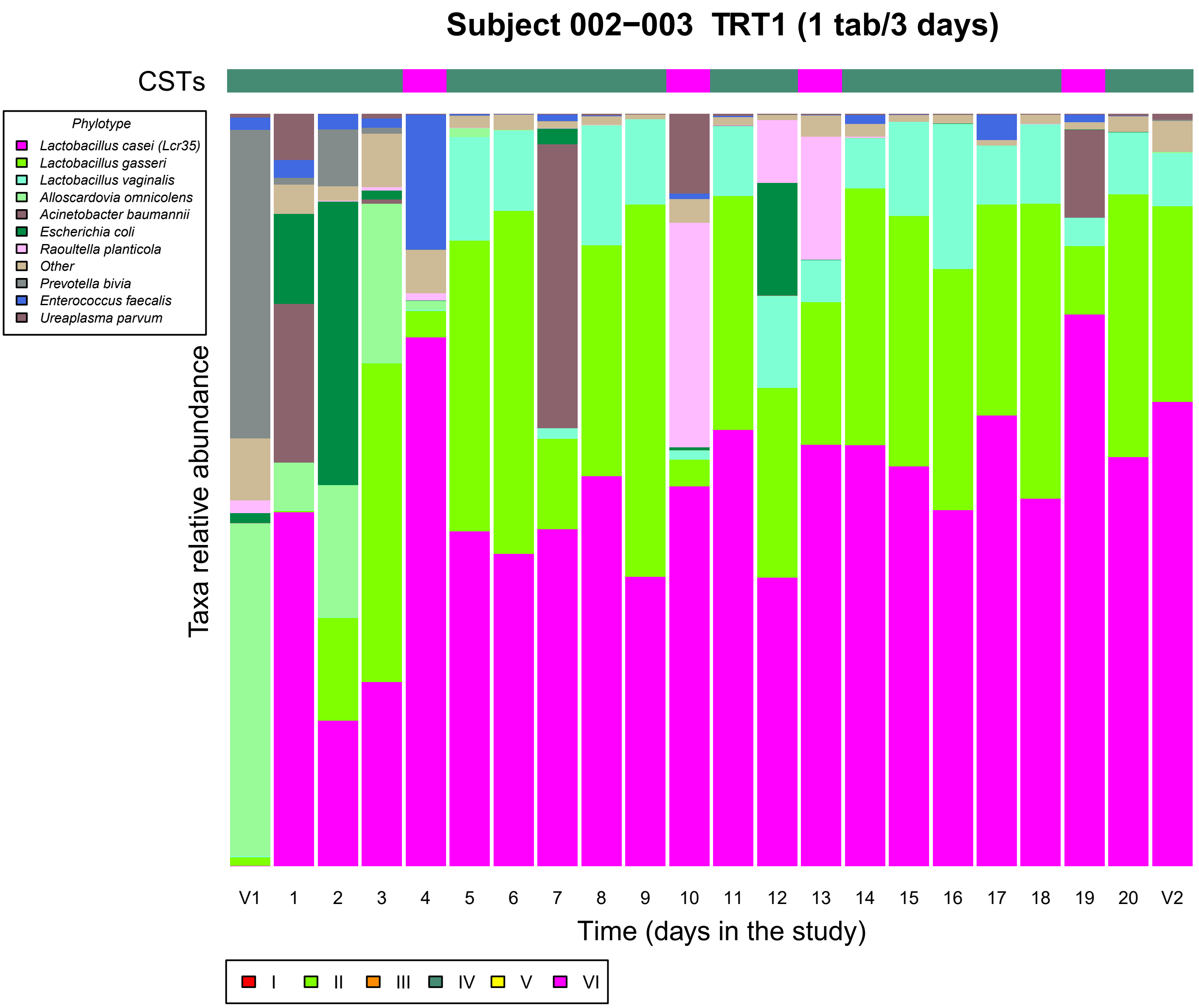

Subject 002−003 TRT1 (1 tab/3 days)
CSTs
Phylotype
Lactobacillus casei (Lcr35)
Lactobacillus gasseri
Lactobacillus vaginalis
Alloscardovia omnicolens
Acinetobacter baumannii
Escherichia coli
Raoultella planticola
Other
Prevotella bivia
Enterococcus faecalis
Ureaplasma parvum
Taxa relative abundance
V1
1
2
3
4
5
6
7
8
9
10
11
12
13
14
15
16
17
18
19
20
V2
Time (days in the study)
I
II
III
IV
V
VI

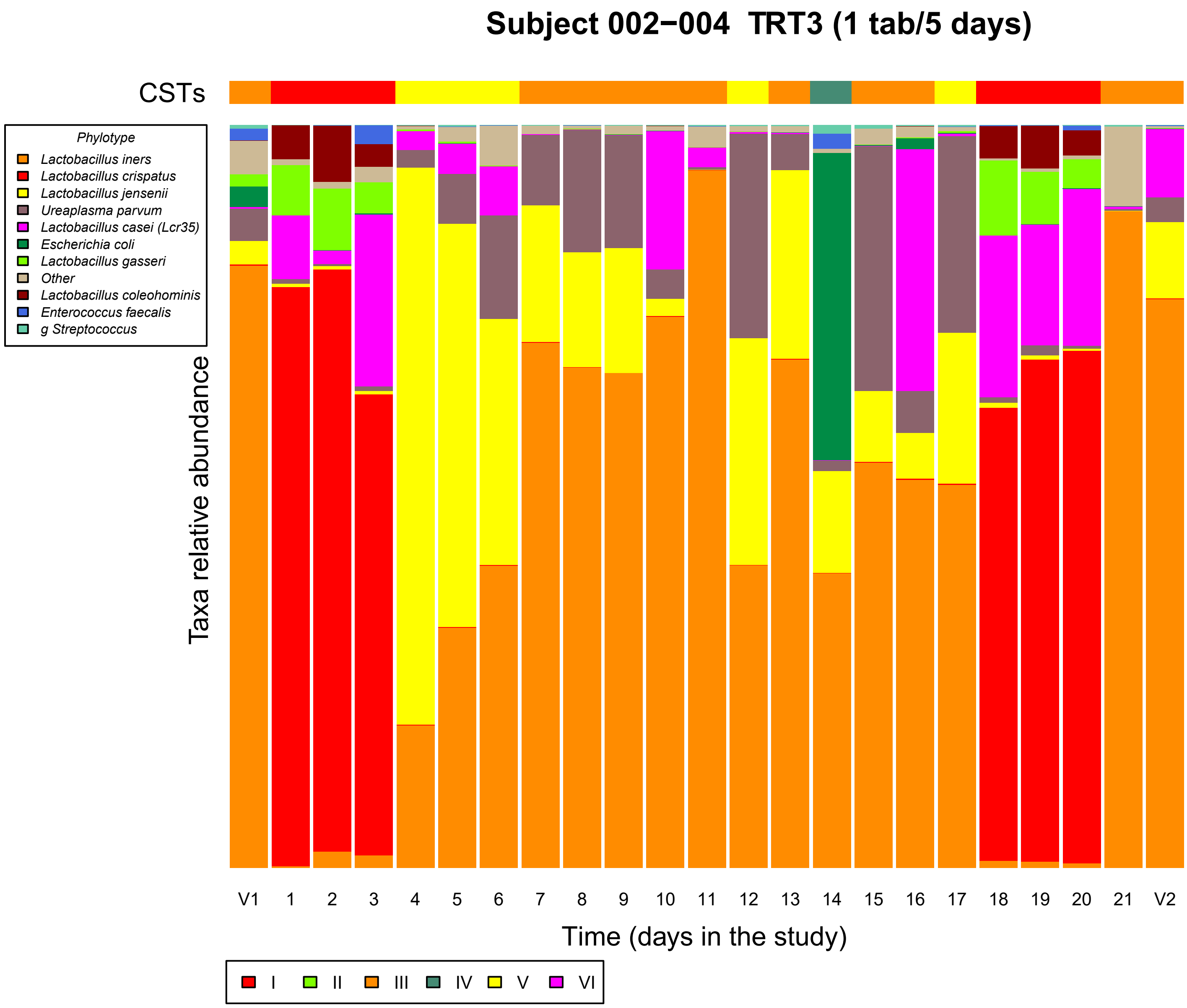
Subject 002−004 TRT3 (1 tab/5 days)
CSTs
Phylotype
Lactobacillus iners
Lactobacillus crispatus
Lactobacillus jensenii
Ureaplasma parvum
Lactobacillus casei (Lcr35)
Escherichia coli
Lactobacillus gasseri
Other
Lactobacillus coleohominis
Enterococcus faecalis
g Streptococcus
Taxa relative abundance
V1 1 2 3 4 5 6 7 8 9 10 11 12 13 14 15 16 17 18 19 20 21 V2
Time (days in the study)
I II III IV V VI

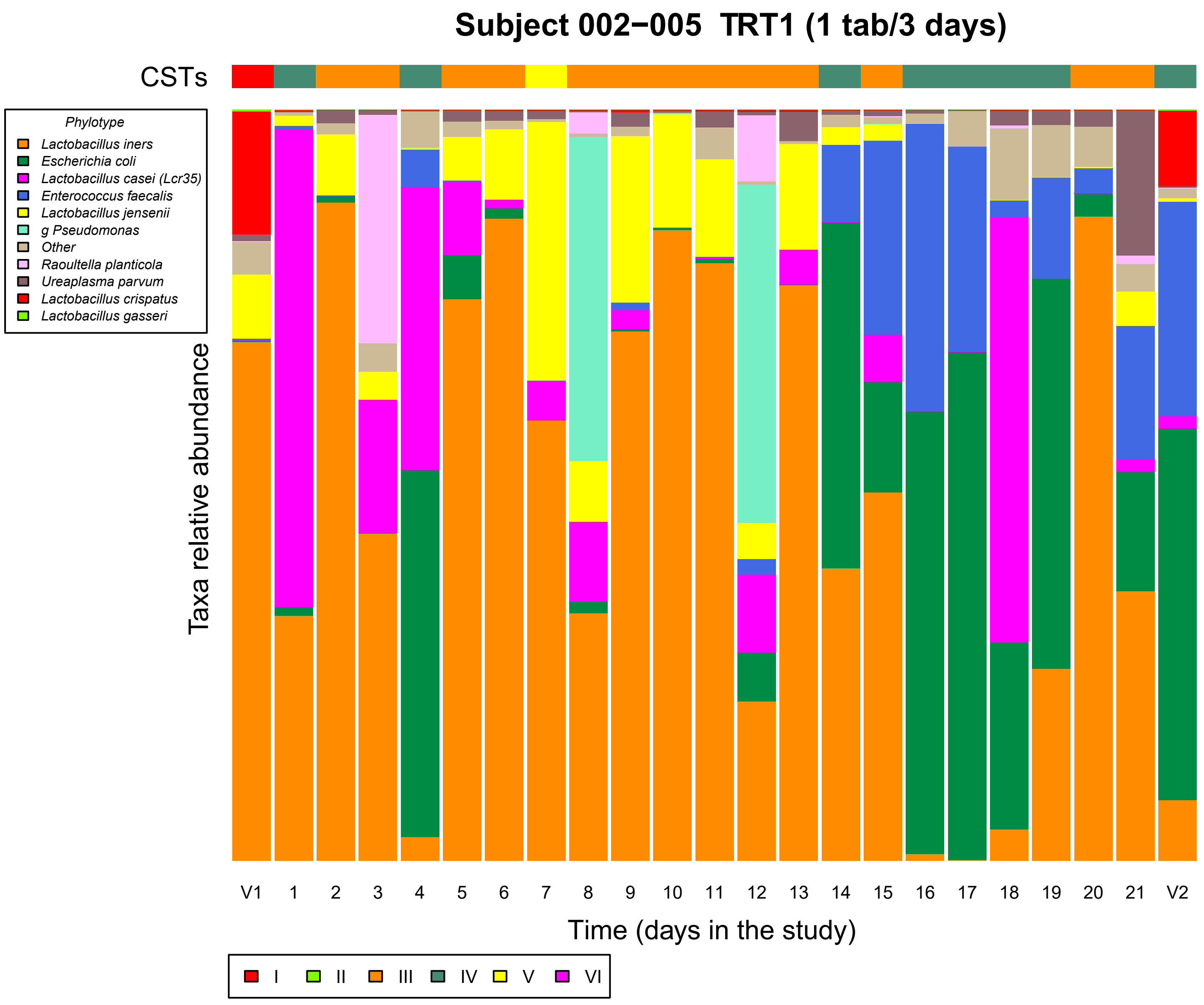

Subject 002−005 TRT1 (1 tab/3 days)
CSTs
Phylotype
Lactobacillus iners
Escherichia coli
Lactobacillus casei (Lcr35)
Enterococcus faecalis
Lactobacillus jensenii
g Pseudomonas
Other
Raoultella planticola
Ureaplasma parvum
Lactobacillus crispatus
Lactobacillus gasseri
Taxa relative abundance
V1 1 2 3 4 5 6 7 8 9 10 11 12 13 14 15 16 17 18 19 20 21 V2
Time (days in the study)
I II III IV V VI

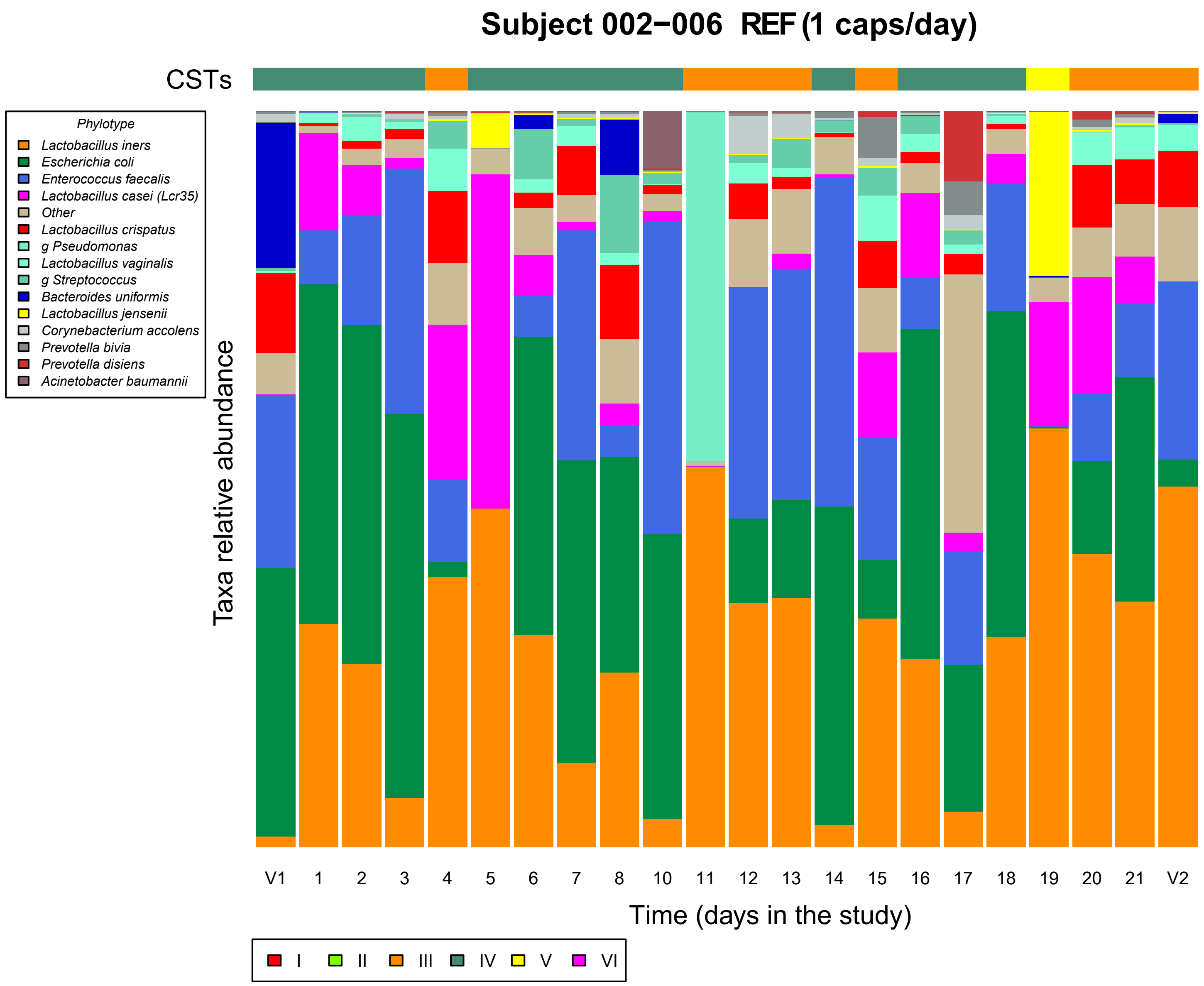

Subject 002−006 REF (1 caps/day)
CSTs
Phylotype
Lactobacillus iners
Escherichia coli
Enterococcus faecalis
Lactobacillus casei (Lcr35)
Other
Lactobacillus crispatus
g Pseudomonas
Lactobacillus vaginalis
g Streptococcus
Bacteroides uniformis
Lactobacillus jensenii
Corynebacterium accolens
Prevotella bivia
Prevotella disiens
Acinetobacter baumannii
Taxa relative abundance
V1 1 2 3 4 5 6 7 8 10 11 12 13 14 15 16 17 18 19 20 21 V2
Time (days in the study)
I II III IV V VI

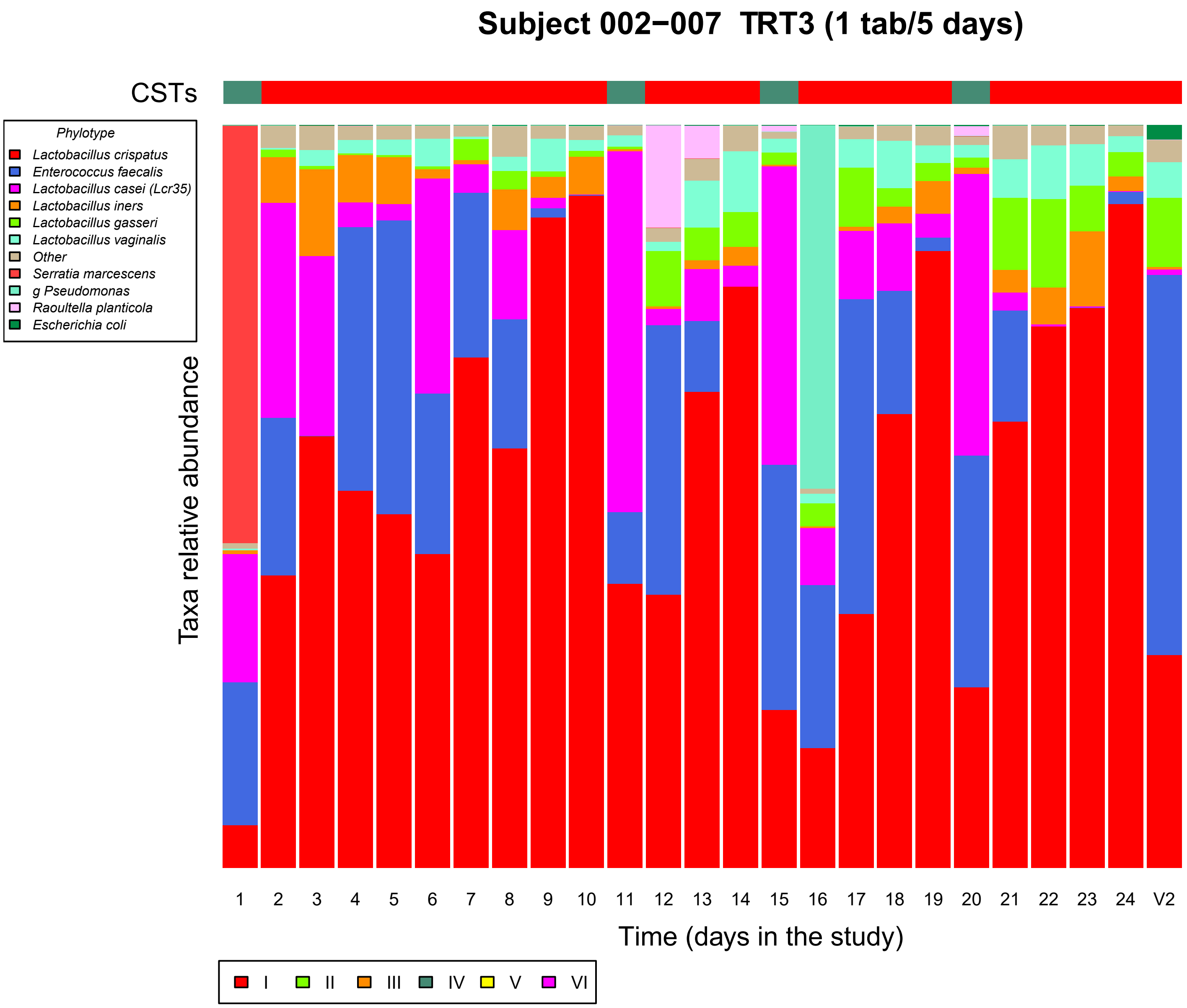

Subject 002−007 TRT3 (1 tab/5 days)
CSTs
Phylotype
Lactobacillus crispatus
Enterococcus faecalis
Lactobacillus casei (Lcr35)
Lactobacillus iners
Lactobacillus gasseri
Lactobacillus vaginalis
Other
Serratia marcescens
g Pseudomonas
Raoultella planticola
Escherichia coli
Taxa relative abundance
1 2 3 4 5 6 7 8 9 10 11 12 13 14 15 16 17 18 19 20 21 22 23 24 V2
Time (days in the study)
I II III IV V VI

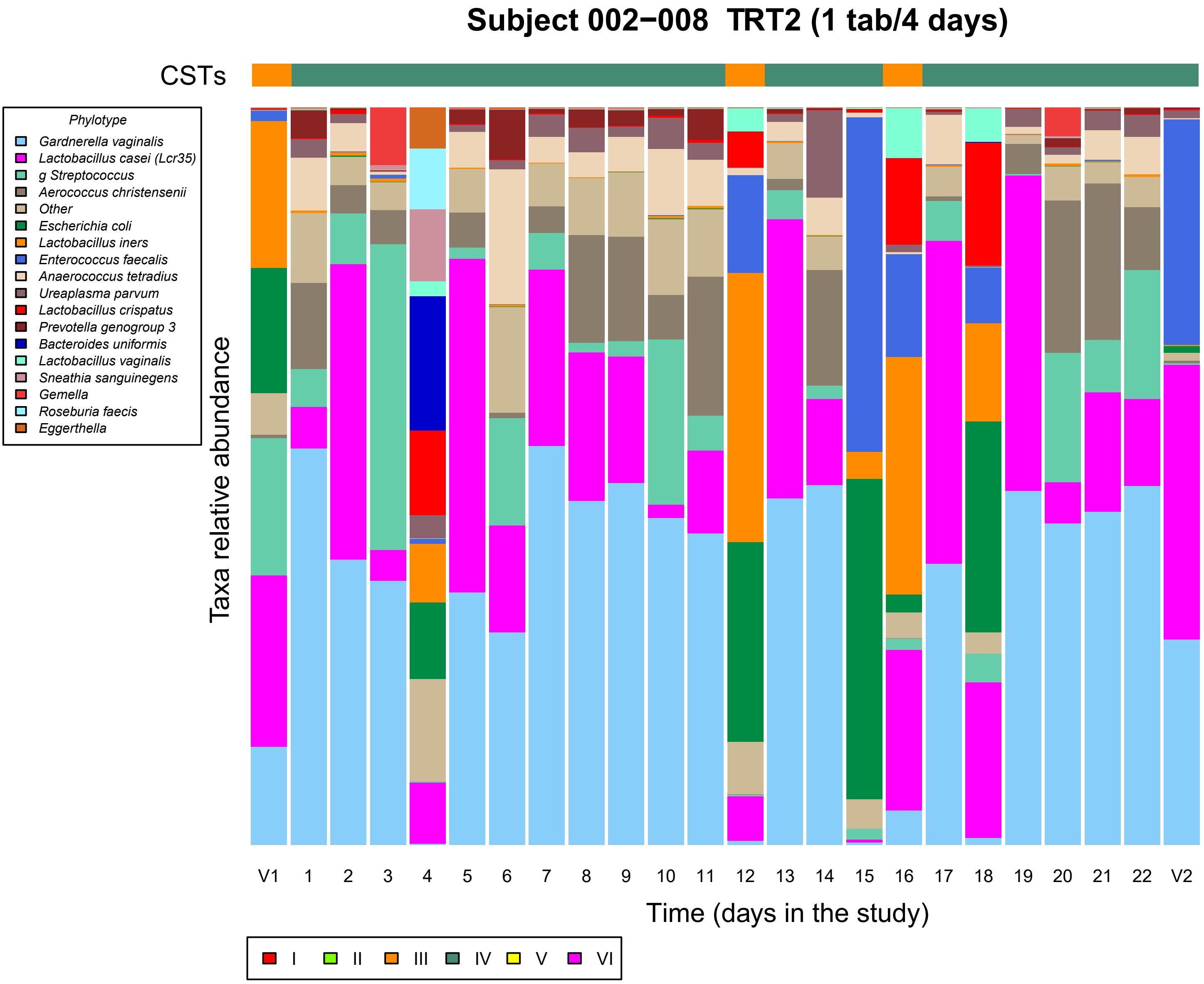

Subject 002−008 TRT2 (1 tab/4 days)
CSTs
Phylotype
Gardnerella vaginalis
Lactobacillus casei (Lcr35)
g Streptococcus
Aerococcus christensenii
Other
Escherichia coli
Lactobacillus iners
Enterococcus faecalis
Anaerococcus tetradius
Ureaplasma parvum
Lactobacillus crispatus
Prevotella genogroup 3
Bacteroides uniformis
Lactobacillus vaginalis
Sneathia sanguinegens
Gemella
Roseburia faecis
Eggerthella
Taxa relative abundance
V1 1 2 3 4 5 6 7 8 9 10 11 12 13 14 15 16 17 18 19 20 21 22 V2
Time (days in the study)
I II III IV V VI

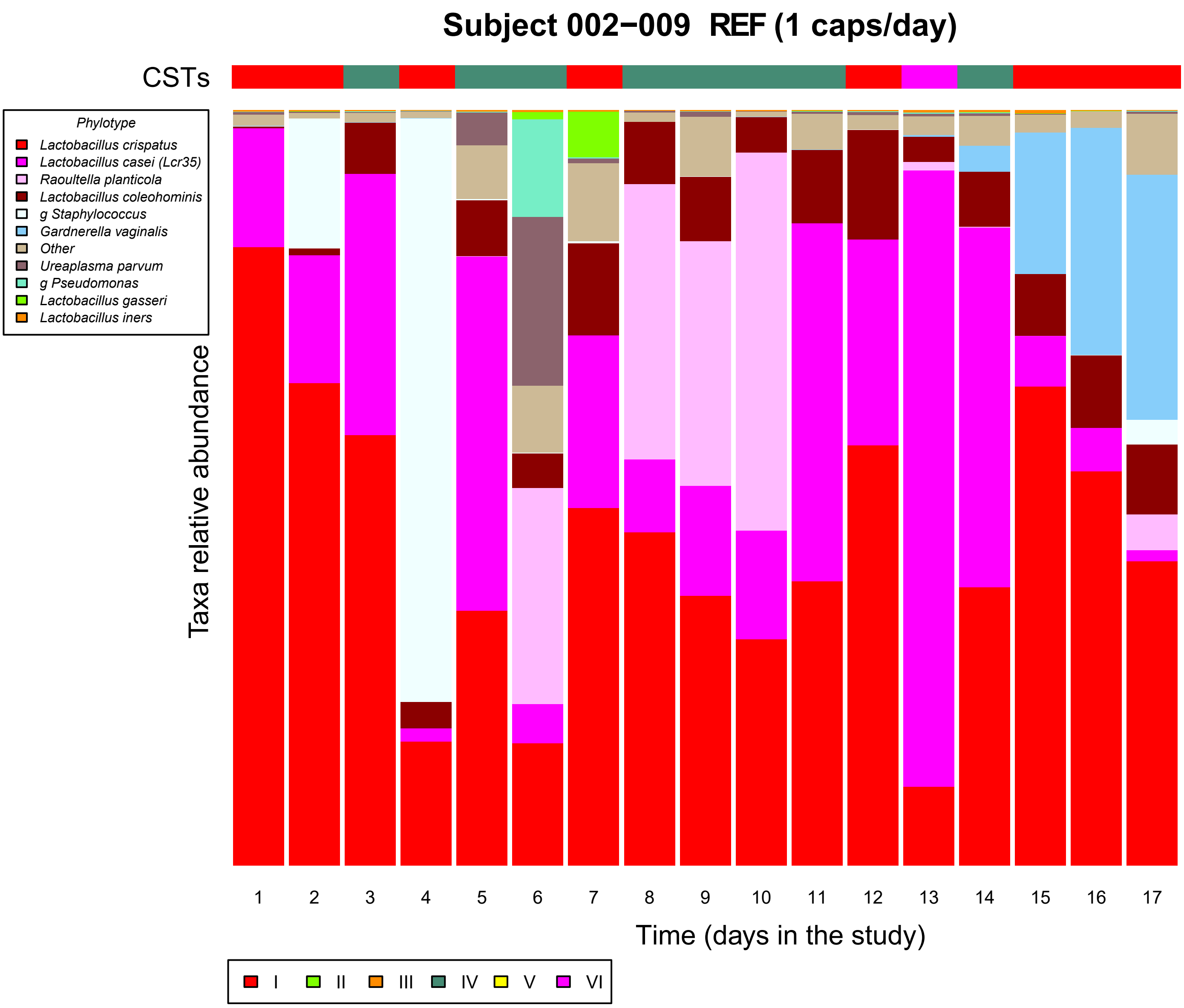
Subject 002−009 REF (1 caps/day)
CSTs
Phylotype
Lactobacillus crispatus
Lactobacillus casei (Lcr35)
Raoultella planticola
Lactobacillus coleohominis
g Staphylococcus
Gardnerella vaginalis
Other
Ureaplasma parvum
g Pseudomonas
Lactobacillus gasseri
Lactobacillus iners
Taxa relative abundance
1 2 3 4 5 6 7 8 9 10 11 12 13 14 15 16 17
Time (days in the study)
I II III IV V VI

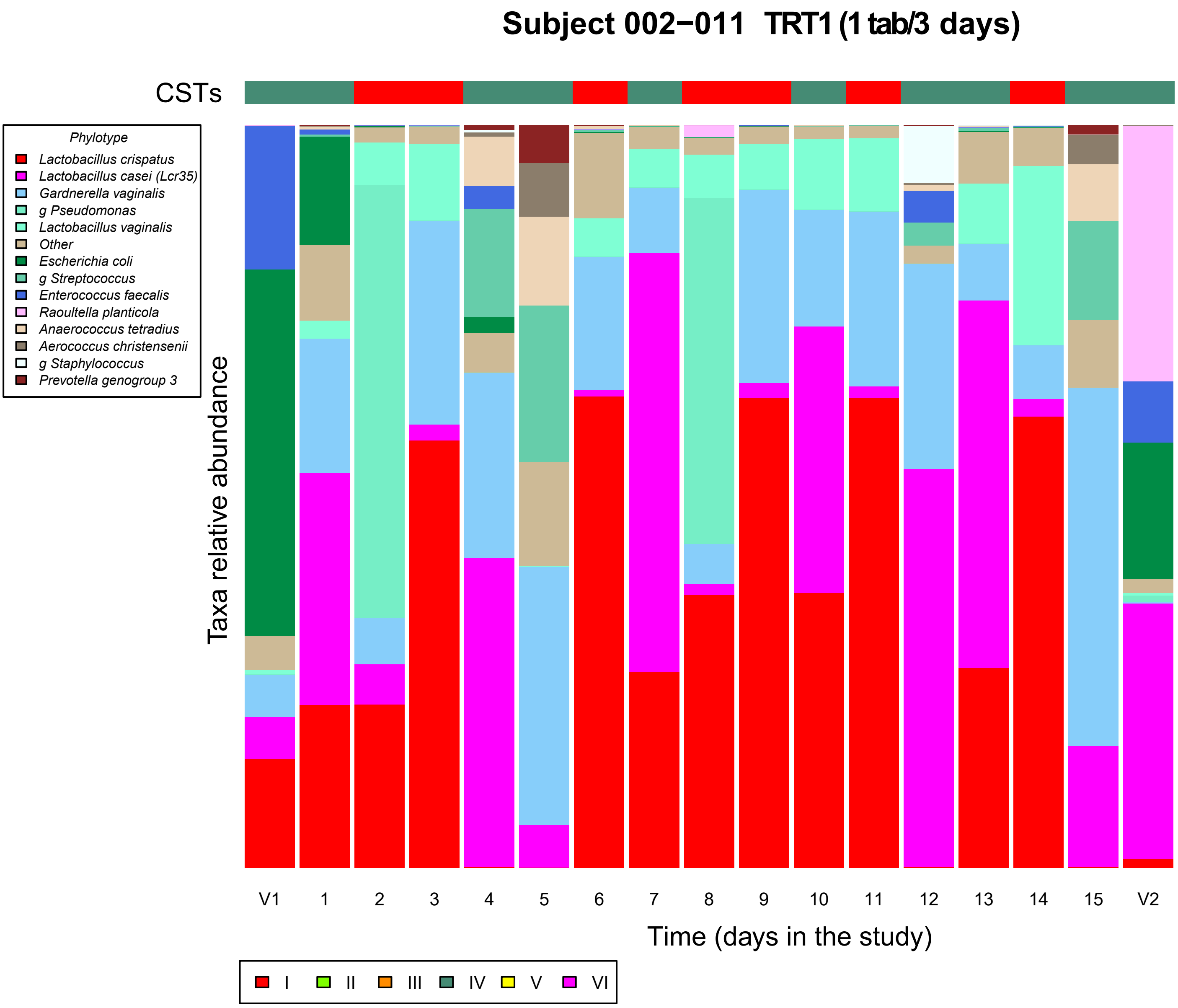
Subject 002−011 TRT1 (1 tab/3 days)
CSTs
Phylotype
Lactobacillus crispatus
Lactobacillus casei (Lcr35)
Gardnerella vaginalis
g Pseudomonas
Lactobacillus vaginalis
Other
Escherichia coli
g Streptococcus
Enterococcus faecalis
Raoultella planticola
Anaerococcus tetradius
Aerococcus christensenii
g Staphylococcus
Prevotella genogroup 3
Taxa relative abundance
V1 1 2 3 4 5 6 7 8 9 10 11 12 13 14 15 V2
Time (days in the study)
I II III IV V VI

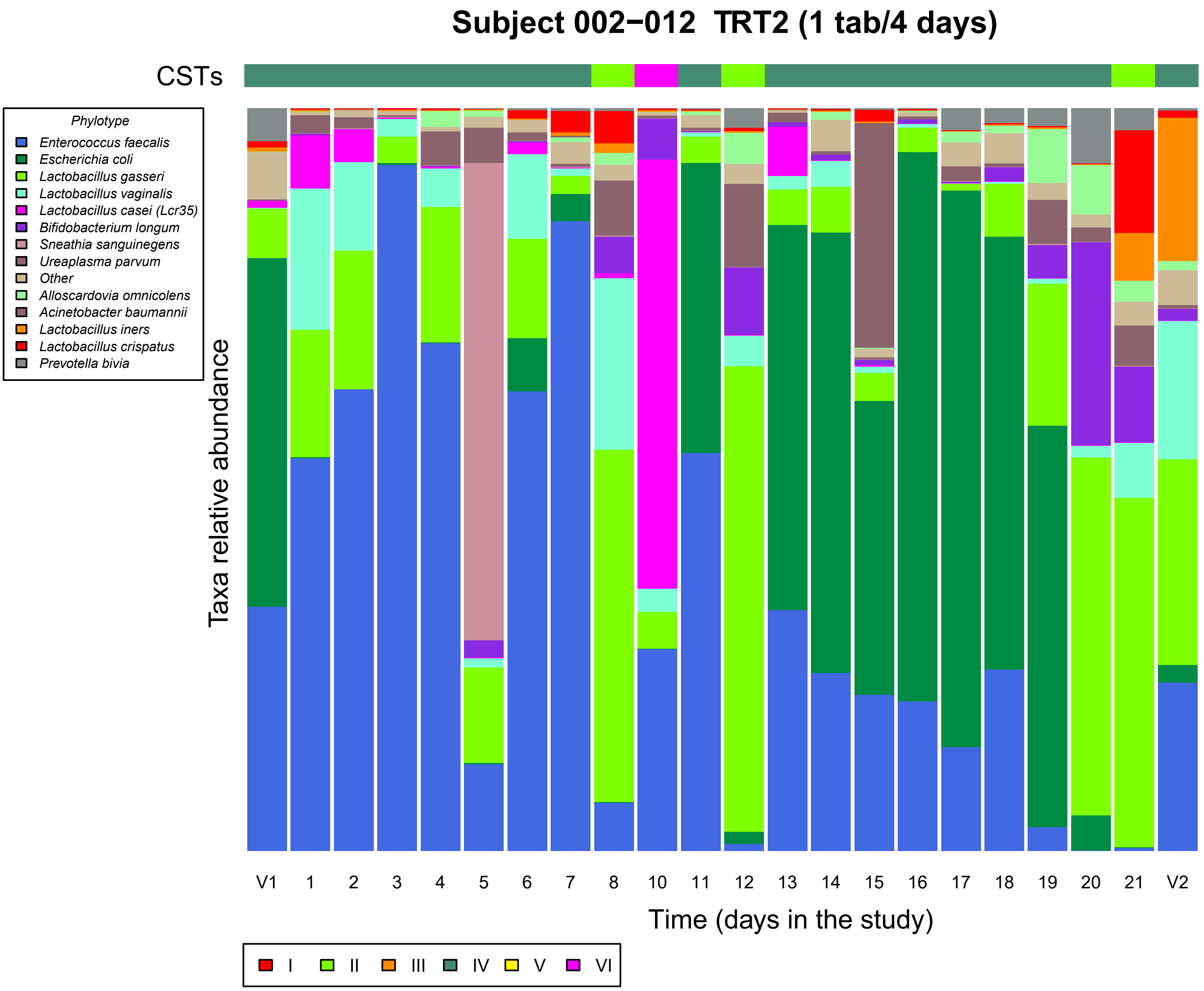

Subject 002−012 TRT2 (1 tab/4 days)
CSTs
Phylotype
Enterococcus faecalis
Escherichia coli
Lactobacillus gasseri
Lactobacillus vaginalis
Lactobacillus casei (Lcr35)
Bifidobacterium longum
Sneathia sanguinegens
Ureaplasma parvum
Other
Alloscardovia omnicolens
Acinetobacter baumannii
Lactobacillus iners
Lactobacillus crispatus
Prevotella bivia
Taxa relative abundance
V1 1 2 3 4 5 6 7 8 10 11 12 13 14 15 16 17 18 19 20 21 V2
Time (days in the study)
I II III IV V VI
